# Attitude Stabilization of Spacecraft in Very Low Earth Orbit by Center-Of-Mass Shifting

**DOI:** 10.3389/frobt.2019.00007

**Published:** 2019-02-14

**Authors:** Josep Virgili-Llop, Halis C. Polat, Marcello Romano

**Affiliations:** Spacecraft Robotics Laboratory, Naval Postgraduate School, Monterey, CA, United States

**Keywords:** spacecraft aerodynamics, attitude stabilization, Very Low Earth Orbit, attitude control, shifting masses, movable masses, CubeSat, aerodynamic disturbance

## Abstract

At very low orbital altitudes (≲450 km) the aerodynamic forces can become major attitude disturbances. Certain missions that would benefit from a very low operational altitude require stable attitudes. The use of internal shifting masses, actively shifting the location of the spacecraft center-of-mass, thus modulating, in direction and magnitude, the aerodynamic torques, is here proposed as a method to reject these aerodynamic disturbances. A reduced one degree-of-freedom model is first used to evaluate the disturbance rejection capabilities of the method with respect to multiple system parameters (shifting mass, shifting range, vehicle size, and altitude). This analysis shows that small shifting masses and limited shifting ranges suffice if the nominal center-of-mass is relatively close to the estimated center-of-pressure. These results are confirmed when the analysis is extended to a full three rotational degrees-of-freedom model. The use of a quaternion feedback controller to detumble a spacecraft operating at very low altitudes is also explored. The analysis and numerical simulations are conducted using a nonlinear dynamic model that includes the full effects of the shifting masses, a realistic atmospheric model, and uncertain spacecraft aerodynamic properties. Finally, a practical implementation on a 3U CubeSat using commercial-off-the-shelf components is briefly presented, demonstrating the implementation feasibility of the proposed method.

## 1. Introduction

Lowering the operational altitude of Earth observation spacecraft can increase the overall cost-effectiveness of a space system (Shao et al., 2014). For example, by lowering the operational altitude, the resolution of a given optical instrument, its radiometric performance and the geospatial accuracy of its imagery are improved. For radar payloads, either the antenna size or the transmission power can be reduced. Furthermore, a given launcher can usually deliver more payload at lower altitude orbits or, for a given spacecraft mass, a less capable and potentially more cost-effective launcher can be used (Virgili-Llop, 2014; Virgili-Llop et al., 2014a).

Lowering the operational altitude forces spacecraft to orbit through denser regions of the atmosphere. The interaction of spacecraft with the residual atmosphere results in aerodynamic forces that, at low altitudes, can become major orbit and attitude perturbations (Fortescue and Stark, 1995). As the aerodynamic forces are only dominant in the lower part of the Low Earth Orbit (LEO) range the term Very Low Earth Orbit (VLEO) is used in this paper to make clear that the considered orbit range extends only up to ~450 km in altitude (Virgili-Llop, 2014; Virgili-Llop et al., 2014a).

Although these aerodynamic perturbations are usually perceived as drawbacks, they can also be seen as an advantage (Virgili-Llop, 2014; Virgili-Llop et al., 2014a). As the orbital lifetime is reduced, there is no need to de-orbit spacecraft at the end of their operational life. Space debris also decay at a faster rate and thus do not accumulate at the same pace in VLEO, reducing the collision risk and greatly increasing the required object density to generate a Kessler syndrome runaway (Wertz et al., 2012). Additionally, the aerodynamic forces can be harnessed to control the spacecraft's orbit (Bevilacqua and Romano, 2008; Virgili-Llop et al., 2014b; Virgili et al., 2015) and attitude (Kumar et al., 1995, 1996; Psiaki, 2004; Gargasz, 2007; Guettler, 2007).

Many missions that would benefit from lower operational altitudes also require a constant and stable attitude (e.g., geodesy spacecraft, Earth observation). In such missions, the attitude perturbations caused by the aerodynamic forces need to be eliminated. An effective and conceptually simple measure to reduce the aerodynamic forces for a given operational altitude is to minimize the spacecraft's cross section area exposed to the incident flow. For this very reason, spacecraft operating in VLEO tend to be slender (Bowman and Lewis, 2002; Drinkwater et al., 2007). To minimize the attitude perturbation it is also highly desirable to design spacecraft with their Center-of-Mass (CoM) as close as possible to the spacecraft's Center-of-Pressure (CoP), thus minimizing the force lever arm and ultimately reducing the aerodynamic disturbance torque. Unfortunately, spacecraft aerodynamics uncertainties (Moe and Moe, 2010; Prieto et al., 2014) and atmospheric variability (Larsen and Fesen, 2009; Pardini et al., 2012) introduce uncertainties and variability to the CoP location. Additional practical design constrains on the location of the CoM make the realization of an overlapping CoP and CoM impossible in practice. A residual aerodynamic disturbance torque will remain and will need to be rejected.

These attitude perturbations can be compensated for by using traditional attitude control actuators (such as reaction wheels). At VLEO the aerodynamic disturbances can be significant and can present a secular component that can quickly saturate momentum exchange devices. In this paper, we explore the use of internal shifting masses as a method to control and ultimately reject these undesired aerodynamic disturbances. The set of internal shifting internal masses, actively change the spacecraft's CoM location, modulating, in direction and magnitude, the aerodynamic torques. Specifically, we are interested in: devising control methods to drive the shifting masses, evaluating the disturbance rejection capabilities under realistic conditions, and evaluating the implementation feasibility of the whole shifting masses concept.

The use of shifting masses as attitude control actuators has already been proposed in the past to help detumble spacecraft (Edwards and Kaplan, 1974; Kunciw and Kaplan, 1976), control the coning motion of a spinning spacecraft (Hamidi-Hashemi, 1993; Halsmer and Mingori, 1995; Janssens and van der Ha, 2014), control the pitch and yaw of solar-sails (Wie, 2004; Wie and Murphy, 2007; Scholz et al., 2011) and, in general, to complement traditional attitude control actuators (Kumar, 2010; Ahn, 2012; Atkins and Henderson, 2012).

Of particular interest is the work by Chesi et al. (2017) who proposes the use of aerodynamic drag to generate attitude control torques modulated in magnitude and direction by actively shifting a set of internal masses. Although Chesi's work, simplifies the effects of the shifting masses on the spacecraft dynamics, ignores the variable and unpredictable nature of the Earth's atmosphere, and assumes that the aerodynamic properties are known and constant, it shows the conceptual feasibility of using shifting masses to control the aerodynamic torques. In particular, it shows that by using a set of three shifting masses augmented by reaction wheels or magnetic torquers and using an adaptive non-linear feedback control law, a spacecraft could be slowly brought, from any initial attitude and angular velocity, to a desired attitude whilst minimizing the use of the reaction wheels or magnetic torquers.

The work presented in this paper takes Chesi's concept one step forward by dropping the dynamic model simplifications, introducing uncertainties into the aerodynamic properties, and adding atmospheric variability. Additional contributions of the work presented in this paper are a sensitivity analysis of the method's performance with respect to the CoP to CoM distance, size of the spacecraft, and operating altitude. Additionally, an assessment of the implementation feasibility of the concept is briefly presented. As in Chesi et al. (2017), the assumptions used to derive the dynamic model and the controllers are made explicit throughout the paper and are marked with *Asm*.

This paper is organized as follows. The spacecraft model is briefly presented in section 2. Then the equations of motion of a spacecraft with internal moving parts are derived in section 3. The uncertain nature of the aerodynamic disturbance caused by a variable atmosphere and the uncertain aerodynamics is subsequently presented in section 4. A reduced one rotational degree-of-freedom model with one shifting mass driven by a Proportional-Integral-Derivative (PID) controller is derived in section 5. This PID controller is used to analyze the disturbance rejection capabilities of the system with respect to several parameters (shifting mass, shifting range, operating altitude and vehicle size). Then we use a full three degrees-of-freedom model with two shifting masses driven by a Linear Quadratic Regulator (LQR) based controller moving along the pitch and yaw axes and augmented by an ideal actuator in roll in section 6 to confirm that the results obtained in the one rotational degree-of-freedom reduced model also apply in a three degrees-of-freedom model. Then, the detumbling capabilities of the proposed method are briefly explored in section 6 with a quaternion feedback controller. Finally, a practical implementation, only using Commercial-Off-The-Shelf (COTS) components, on a 3U CubeSat is presented in section 7.

## 2. Spacecraft Model

To keep the analysis as general as possible, a spherically shaped spacecraft has been used. Although a spheric spacecraft may be initially perceived as a simplistic case, it can already be used to illustrate the effects of aerodynamic uncertainties without dwelling into more complex shapes. Also, the simple relationship between the size of the sphere (its radius) and its mass properties (inertia and mass) is used to extract the general trends with respect to spacecraft size.

- *Asm.1*: The spacecraft is spherically shaped.

**Figure 1:**
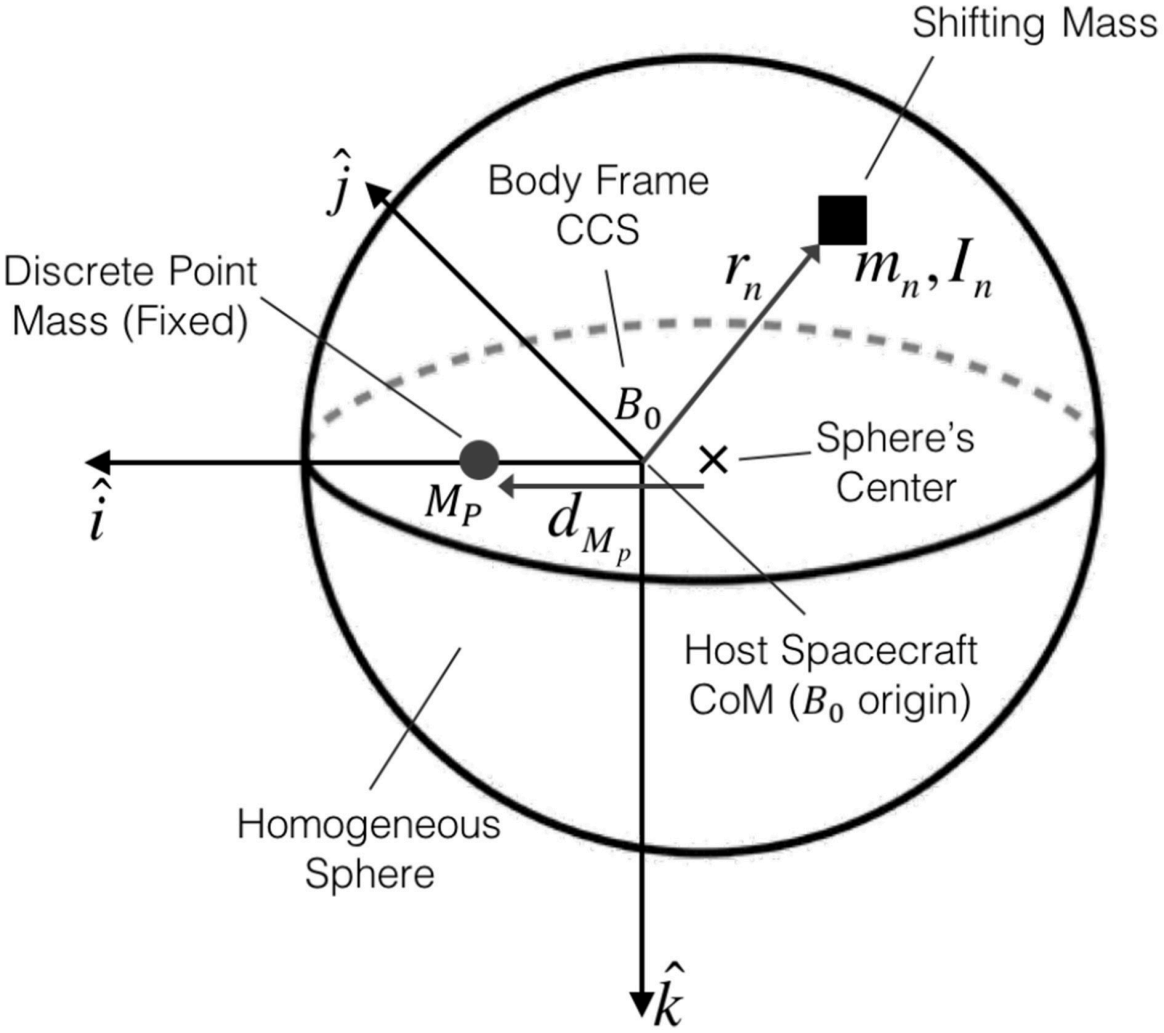
Spacecraft model with its homogeneous sphere, the discrete point mass along the $\hat{i}$ axis and a generic shifting mass *m*_*n*_.

The spherically shaped spacecraft hosting the shifting masses (host spacecraft) is assumed to be composed of a homogeneous density sphere and a fixed discrete point mass (not a shifting mass) as shown in Figure 1. The discrete point mass is added to the host vehicle to obtain a host spacecraft CoM that is not coincident with the sphere's geometric center. It is worth mentioning at this point that the shifting masses are not part of the host spacecraft. Excluding the shifting masses greatly simplifies the equations of motion as it is shown in section 2.

The mass of a homogeneous density sphere is $M_{S}=\rho_{S}4/3\pi R^{3}$ and its inertia $J_{S}=I\rho_{s}8/15\pi R^{5}$, with *ρ*_*S*_ denoting the sphere's density, *R* the sphere's radius, and ***I*** the identity matrix. The mass of the fixed discrete point mass *M*_*P*_ can be expressed as a fraction κ of the homogeneous sphere's mass *M*_*P*_ = κ*M*_*S*_. The total mass of the host vehicle *M*_0_ is simply *M*_0_ = *M*_*S*_(1 + κ). Let the host spacecraft's CoM define the origin of the spacecraft's body frame *B*_0_ with the discrete point mass located along the $\hat{i}$ axis, as depicted in Figure 1.

The goal of the attitude control system is to keep the attitude of the spacecraft stable with respect to the orbital frame (${\hat{k}}_{\text{orbit}}$ points nadir, ${\hat{i}}_{\text{orbit}}$ along the inertial velocity vector, and ${\hat{j}}_{\text{orbit}}$ completes the right hand triad). In this case, the desired attitude will be to align the axes of the body frame *B*_0_ with the axes of the orbital frame, thus in the desired attitude $\hat{i}$ represents the roll axis, $\hat{j}$ the pitch axis, and $\hat{k}$ the yaw axis.

As the location of the discrete point mass is artificially restricted to the $\hat{i}$ axis, the desired attitude represents an aerodynamic equilibrium attitude (in the absence of wind and atmospheric co-rotation). The implications of this assumption will become clear in section 4.4, but briefly stated, by selecting an aerodynamic equilibrium attitude we avoid secular aerodynamic torques. If an arbitrary attitude was selected the location of the shifting masses would be biased in order to provide this secular torque (essentially moving the system's CoM and forcing the desired attitude to become an equilibrium one).

- *Asm.2*: The desired attitude is an aerodynamic equilibrium attitude (in the absence of wind and atmospheric co-rotation).

The inertia properties of the host spacecraft ***J***_0_ can be computed using Equation (1), with the distance between the point mass and the sphere's center denoted by *d*_*M*_*P*__. If *d*_*M*_*P*__ > 0 the CoM will be located in the positive side of $\hat{i}$ and if *d*_*M*_*P*__ < 0 then the CoM will be in the negative side of $\hat{i}$. The location of the host spacecraft's CoM from the sphere center is simply $d_{\text{Co}{\text{M}}_{0}}=\frac{\kappa d_{M_{P}}}{\left(1+\kappa \right)}$.

(1) $$\begin{array}{l} J_{0}=J_{S}+\frac{M_{S}\left(1\;+\;\kappa \right)}{\kappa }\left[\begin{array}{ccc} 0 & 0 & 0\\ 0 & d_{M_{P}}^{2} & 0\\ 0 & 0 & d_{M_{p}}^{2} \end{array}\right] \end{array}$$

Note that the host spacecraft is symmetric with respect to the roll axis $\hat{i}$ and thus the definition of the pitch $\hat{j}$ and yaw $\hat{k}$ axes is arbitrary.

To this host vehicle, whose mass and inertia properties are fixed, known and constant, a set of *N* shifting masses *m*_*n*_ is added, altering the mass, inertia, and CoM location of the resulting combined system. As the body reference frame is defined with respect to the CoM of the host spacecraft, excluding the shifting masses, the system's CoM, including the shifting masses, will be variable and thus will not be located at the origin of the body reference frame *B*_0_.

## 3. Dynamic Model

The equations of motion for a system of connected rigid bodies is derived in this section. These equations of motion, which take into account the dynamic effects of the shifting masses, are used during the numerical simulations and serve as the starting dynamic model used to derive the controllers. The following assumptions are made to simplify the formulation of the dynamics.

- *Asm.3*: The host spacecraft is a rigid body.
- *Asm.4*: The shifting masses are rigid bodies.

Under these assumptions, the fundamental equation describing the rotational motion of a system of connected rigid bodies is given by Equation (2) (Grubin, 1962; Edwards and Kaplan, 1974).

(2) $$\begin{array}{l} \tau =\dot{H}+S\times a \end{array}$$

In Equation (2) ***τ*** denotes the external torques around an arbitrary reference point, $\dot{H}$ the time derivative of the system's angular momentum around this reference point, ***S*** the system's first moment of mass with respect to the reference point, and ***a*** denotes the inertial acceleration of the reference point. The system's reference point can be arbitrarily selected and can move in an arbitrary manner. It is interesting to note that if the acceleration of the reference point is ***a*** = 0 or if the reference point is selected as the system's CoM (***S*** = 0) then the usual expression $\tau =\dot{H}$ is recovered.

As introduced in the previous section, the system is composed of a host vehicle to which *N* shifting masses are added. To simplify the derivation of the equations, and without loosing generality, it will be considered that the reference point of the system is the host vehicle's CoM (which excludes the shifting masses). This reference point is the origin of the body frame *B*_0_ as shown in Figure 1. This assumption is useful, as by definition, the host vehicle's mass *M*_0_ and its inertia ***J***_0_ are constant when projected into *B*_0_. Additionally, the movement of the shifting masses can be easily known with respect to the host spacecraft reference frame *B*_0_.

The *N* shifting masses have their own reference frames *B*_*n*_ with their origin located at the CoM of the shifting mass. The shifting masses can be rigid bodies or point masses. If they are rigid bodies the inertia of the shifting masses in *B*_*n*_ will be denoted as $J_{n}^{B_{n}}$ and when expressed in *B*_0_ it will be simply denoted as ***J***_*n*_.

The location of a shifting mass with respect to the reference point (the origin of *B*_0_) will be denoted as ***r***_*n*_. The ${\dot{r}}_{n}$ and ${\ddot{r}}_{n}$ terms will denote the inertial linear velocity and acceleration of the shifting mass expressed in the *B*_0_ frame. The inertial angular velocity of the host vehicle frame *B*_0_ is denoted by ***ω***_0_ and the term ***ω***_*n*_ denotes the inertial angular velocities of the *B*_*n*_ frames expressed in the *B*_0_ frame.

The inertial angular velocity of the shifting mass can be computed using the following equation, with $\omega_{n}^{\prime }$ being the relative angular velocity of the shifting mass reference *B*_*n*_ with respect to the host vehicle reference *B*_0_.

(3) $$\begin{array}{l} \omega_{n}=\omega_{0}+\omega_{n}^{\prime } \end{array}$$

In such a multibody system, the total angular momentum ***H*** is composed of the sum of the angular momentum of host vehicle ***H***_0_ and of the shifting masses ***H***_*n*_, as shown in Equation (4).

(4) $$\begin{array}{l} H=H_{0}+\overset{N}{\underset{n=1}{\sum }}H_{n} \end{array}$$

(5) $$\begin{array}{l} H_{0}=J_{0}\omega_{0} \end{array}$$

(6) $$\begin{array}{l} H_{n}=J_{n}\omega_{n}+m_{n}r_{n}\times {\dot{r}}_{n} \end{array}$$

The linear inertial velocity of the shifting mass ${\dot{r}}_{n}$ can be simply computed using the transport theorem, resulting in the following equation, where ${\dot{r}}_{n}^{\prime }$ denotes the relative velocity of the shifting mass with respect to *B*_0_.

(7) $$\begin{array}{l} {\dot{r}}_{n}={\dot{r}}_{n}^{\prime }+\omega_{0}\times r_{n} \end{array}$$

To use Equation (2) the angular momentum needs to be differentiated. Deriving (Equation 4) it follows that the total angular momentum time derivative is the sum of the host vehicle and shifting masses angular momentum time derivatives (using the transport theorem where appropriate).

(8) $$\begin{array}{l} \dot{H}={\dot{H}}_{0}+\overset{N}{\underset{n=1}{\sum }}{\dot{H}}_{n} \end{array}$$

(9) $$\begin{array}{l} {\dot{H}}_{0}=J_{0}{\dot{\omega }}_{0}+\omega_{0}\times H_{0} \end{array}$$

(10) $$\begin{array}{l} {\dot{H}}_{n}=J_{n}{\dot{\omega }}_{n}+\omega_{n}\times H_{n}+m_{n}r_{n}\times {\ddot{r}}_{n} \end{array}$$

The inertial acceleration of the shifting masses can be computed with the transport theorem, resulting in the following well known equation.

(11) $$\begin{array}{l} {\ddot{r}}_{n}=\omega_{0}\times \left(\omega_{0}\times r_{n}\right)+{\dot{\omega }}_{0}\times r_{n}+2\omega_{0}\times {\dot{r}}_{n}^{\prime }+{\ddot{r}}_{n}^{\prime } \end{array}$$

Note how ${\dot{r}}_{n}^{\prime }$ and ${\ddot{r}}_{n}^{\prime }$ are the shifting masses relative velocity and acceleration with respect to *B*_0_. These ${\dot{r}}_{n}^{\prime }$ and ${\ddot{r}}_{n}^{\prime }$ magnitudes can be measured inside the host spacecraft.

Moving on with the other terms in Equation (2), the first moment of mass is defined as in the following equation.

(12) $$\begin{array}{l} S=\overset{N}{\underset{n=1}{\sum }}m_{n}r_{n} \end{array}$$

The inertial acceleration of the origin of *B*_0_ (the reference point in Equation 2) can then be written as follows.

(13) $$\begin{array}{l} a={\ddot{r}}_{B_{0}}={\ddot{r}}_{\text{CoM}}-{\ddot{r}}_{\text{CoM}}^{\prime } \end{array}$$

The ${\ddot{r}}_{\text{CoM}}^{\prime }$ term denotes the acceleration of the system's CoM with respect to *B*_0_ (the relative movement of the system's CoM) and ${\ddot{r}}_{\text{CoM}}$ is the inertial acceleration of the system's CoM (due to the external forces ***F***). The ${\ddot{r}}_{\text{CoM}}$ acceleration can be easily computed using Newton's second law and ${\ddot{r}}_{\text{CoM}}^{\prime }$ is obtained by computing the relative CoM acceleration as follows.

(14) $${\overset{..}{r}}_{\text{CoM}}=\frac{F}{M_{0}+\sum_{n=1}^{N}m_{n}}$$

(15) $${\overset{..}{r}}_{\text{CoM}}^{\prime }=\frac{\sum_{n=1}^{N}m_{n}{\overset{..}{r}}_{n}}{M_{0}+\sum_{n=1}^{N}m_{n}}$$

With the equations above, Equation (2) can be fully expanded as in Equation (16).

(16) $$\begin{array}{l} J_{0}{\dot{\omega }}_{0}+\omega_{0}\times J_{0}\omega_{0}+\sum_{n=1}^{N}J_{n}{\dot{\omega }}_{n}+\sum_{n=1}^{N}\omega_{n}\times \left(J_{n}\omega_{n}+m_{n}r_{n}\times {\dot{r}}_{n}\right)\\ \;+\sum_{n=1}^{N}\left(m_{n}r_{n}\times {\overset{..}{r}}_{n}\right)+......+\frac{1}{M_{0}+\sum_{n=1}^{N}m_{n}}\left(\sum_{n=1}^{N}m_{n}{\overset{..}{r}}_{n}\right)\\ \;\times \sum_{n=1}^{N}m_{n}r_{n}=\tau +\frac{F}{M_{0}+\sum_{n=1}^{N}m_{n}}\times \sum_{n=1}^{N}m_{n}r_{n} \end{array}$$

It is interesting to note that (Equation 16) also contains the case of momentum exchange devices (reaction wheels and control moment gyroscopes) and thus these devices can be easily incorporated into this analysis.

The aerodynamic effects on the attitude dynamics of the system are represented by the external torques ***τ*** and forces ***F***. It is important to note that the torques ***τ*** are computed around the fixed reference point (the host vehicle CoM) and not with respect to the moving system's CoM. The term in Equation (16) that contains the external forces ***F*** accommodates this difference. It is also important to note that (Equation 16) contains several terms that depend on the shifting masses relative velocities and accelerations, thus accommodating the dynamic effects of the shifting masses.

### 3.1. Point Mass Simplification

A very useful simplification is obtained when it is assumed that the shifting masses are point masses and do not posses inertia ***J***_*n*_ = 0. In such a case, the rotation of the shifting masses is irrelevant and their translation is the only parameter that affect the dynamics. Under such assumption the general equations of motion (Equation 16) can be simplified as in Equation (17).

(17) $$\begin{array}{l} J_{0}{\dot{\omega }}_{0}+\omega_{0}\times J_{0}\omega_{0}+\sum_{n=1}^{N}\left(m_{n}r_{n}\times {\overset{..}{r}}_{n}\right)\\ \;+\frac{1}{M_{0}+\sum_{n=1}^{N}m_{n}}\left(\sum_{n=1}^{N}m_{n}{\overset{..}{r}}_{n}\right)\times \sum_{n=1}^{N}m_{n}r_{n}=......\\ \;=\tau +\frac{F}{M_{0}+\sum_{n=1}^{N}m_{n}}\times \sum_{n=1}^{N}m_{n}r_{n} \end{array}$$

For a single point mass and introducing the concept of reduced mass μ (Equation 18), the equation can be further simplified to finally obtain (Equation 19), recovering the expression from Edwards and Kaplan (1974).

(18) $$\begin{array}{l} \mu =\frac{mM_{0}}{M_{0}\;+\;m} \end{array}$$

(19) $$\begin{array}{l} J_{0}{\dot{\omega }}_{0}+\omega_{0}\times J_{0}\omega_{0}+\mu r\times \ddot{r}=\tau +\frac{\mu F}{M_{0}}\times r \end{array}$$

## 4. Aerodynamic Modeling

The residual atmosphere present at orbital altitudes causes spacecraft to experience aerodynamic forces (mainly aerodynamic drag). Orbital decay is the main effect of aerodynamic drag but these aerodynamic forces will also induce aerodynamic torques and thus perturb the spacecraft's attitude.

In general, Equation (20) can be used to compute these aerodynamic forces, where ρ denotes the atmospheric density, *V*_∞_ the relative velocity of the spacecraft with respect to the flow, *A*_ref_ an arbitrary reference area (usually taken as the spacecraft's cross section area), and ***C***_***f***_ the force coefficients (along the three different axes). Special cases of Equation (20) use, instead of the generic force coefficients ***C***_***f***_, the drag *C*_*D*_ (anti velocity), and lift *C*_*L*_ (normal to velocity) coefficients, which leads to the well known drag and lift (Equations 21, 22).

(20) $$\begin{array}{l} F=\frac{1}{2}\rho V_{\infty }^{2}A_{ref}C_{f} \end{array}$$

(21) $$\begin{array}{l} D=\frac{1}{2}\rho V_{\infty }^{2}A_{ref}C_{D} \end{array}$$

(22) $$\begin{array}{l} L=\frac{1}{2}\rho V_{\infty }^{2}A_{ref}C_{L} \end{array}$$

From Equation 20 (or Equations 21, 22) the atmospheric density ρ, the relative velocity with respect to the flow *V*_∞_ and the force coefficients *C*_*D*_ and *C*_*L*_ need to be estimated (using the environment and gas-surface interaction models) before the aerodynamic forces can be computed.

It is worth noting at this point that the atmospheric density approximately increases exponentially with decreasing altitude and thus the aerodynamic forces magnitude will also increase exponentially with decreasing orbital altitude. The aerodynamic disturbance is therefore strongly dependent on the altitude and dominates at very low orbital altitudes.

Ideally, the controller that regulates the shifting masses position would know the direction and magnitude of the relative flow *V*_∞_, the atmospheric density ρ, and the aerodynamic properties of the spacecraft ***C***_*f*_. With this information it would be able to accurately estimate the aerodynamic torque that the spacecraft is experiencing and drive the shifting masses to reject it. Unfortunately, the atmospheric environment is highly variable and poorly predictable (Larsen and Fesen, 2009; Pardini et al., 2012) and spacecraft aerodynamics are not particularly well understood (Moe and Moe, 2010; Prieto et al., 2014). As a consequence, the controller will not be able to obtain accurate estimates of the aerodynamic torque magnitude or direction.

In the numerical simulations conducted in this paper, state of the art atmospheric and spacecraft aerodynamic models (see following sections) are used to obtain what it is assumed to be the truth values. The controller will then estimate these magnitudes using simplified aerodynamics and atmospheric models. This set-up ensures the presence of realistic atmospheric variability and realistic aerodynamic properties, while emulating the uncertainty that a controller will be subjected to.

### 4.1. Atmospheric Density Model

For this study, the NRLMSISE-00 (Picone et al., 2002) atmospheric model is used to estimate the atmospheric density ρ. This specific atmospheric model offers a good balance between model accuracy and computational complexity (ECSS Secretariat, 2008).

- *Asm.5*: The atmosphere density behaves as predicted by the NRLMSISE-00 model.

**Figure 2:**
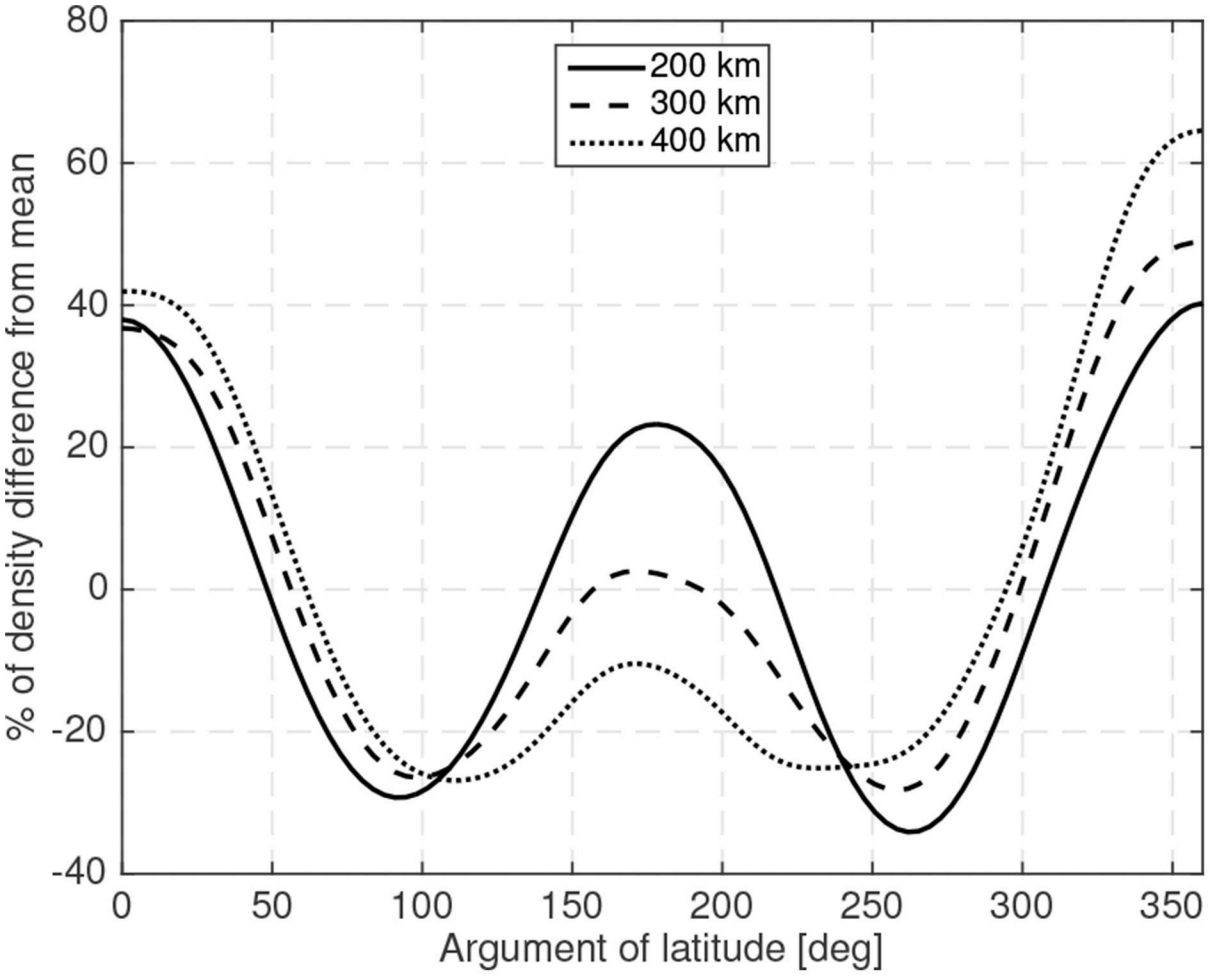
Density variations during a typical circular Sun-synchronous orbit at different operational altitudes.

The Earth's atmosphere not only exhibits vertical density variations but also horizontal ones (as the day-to-night density changes among others). Thus, a spacecraft orbiting in a circular orbit will experience density variations (that will modify the magnitude of the aerodynamic forces). Figure 2 shows the density variations, with respect to the orbit's mean density (using the NRLMSISE-00 model), for a 10:30 Local Time of the Ascending Node (LTAN) circular Sun-synchronous orbit at different orbital altitudes in moderate solar activity (ISO 14222, 2013). Figure 2 exemplifies how variable the density is and thus how variable the magnitude of the drag, and consequently the aerodynamic torque, is during these typical Sun-synchronous orbits.

### 4.2. Wind Model

It is not uncommon to assume that a spacecraft's inertial velocity is equal to the spacecraft's relative velocity with respect to the incoming flow. This assumption ignores that the atmosphere co-rotates with the Earth (Challinor, 1968; King-Hele, 1987, 1992) and that there is atmospheric time-varying wind (Killeen et al., 1982; King-Hele and Walker, 1988). These two effects will make the direction and magnitude of the relative flow *V*_∞_ differ, in direction and magnitude, from the inertial velocity.

**Figure 3:**
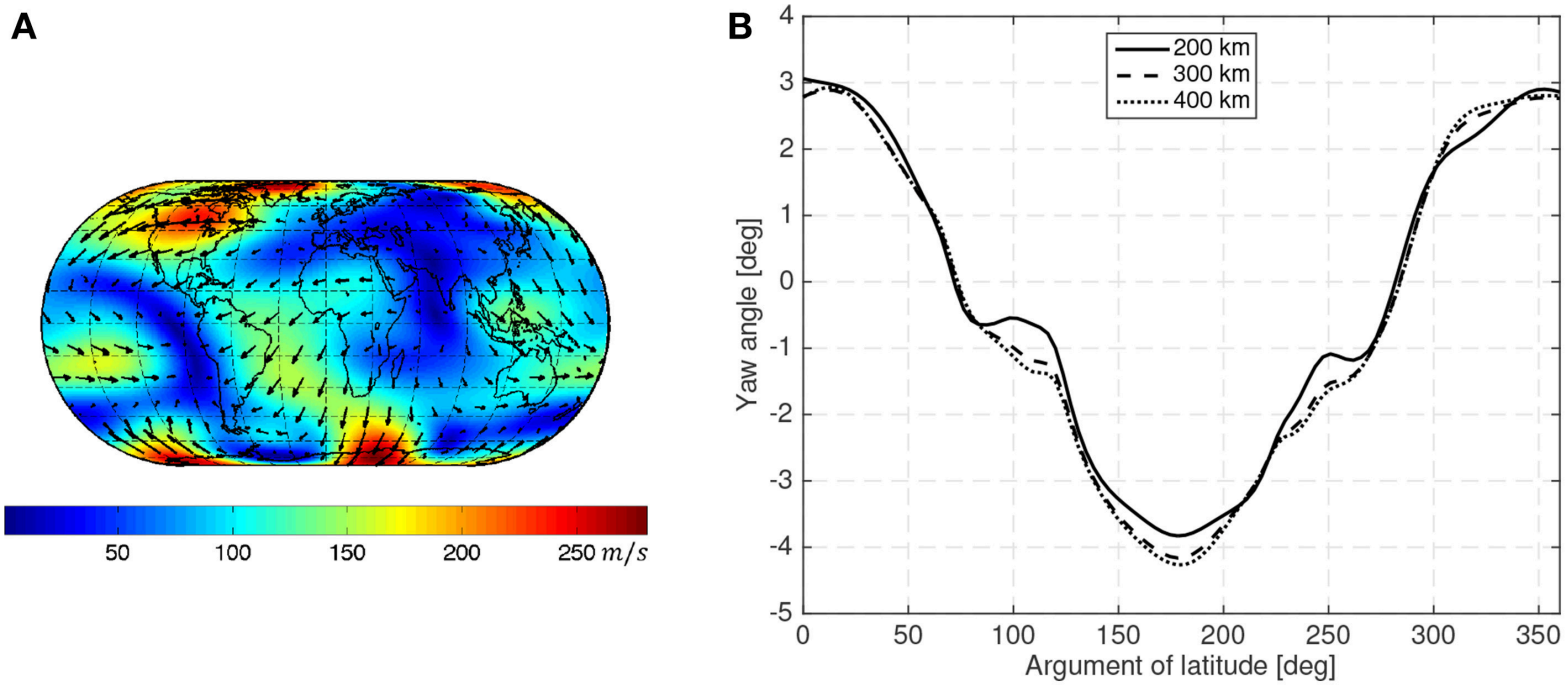
Wind pattern according to the HWM93 (Hedin et al., 1996) model at 450 km with moderate solar activity (ISO 14222, 2013) during northern hemisphere summer solstice **(A)** and yaw angle caused by atmospheric co-rotation and wind during a circular sun-synchronous orbit at different operational altitudes **(B)**.

The atmospheric wind is also highly variable, spatially and temporally. Figure 3A shows an example distribution of the wind. As the atmospheric wind has not been as extensively studied as other atmospheric properties, the existing models are less accurate (Larsen and Fesen, 2009). In this work, the HWM07 (Drob et al., 2008) wind model is used. It has to be noted that this model only provides zonal and meridional wind profiles representative of the climatological averages for various geophysical conditions. Vertical winds, which usually have smaller magnitudes, are not included in the model. Real instantaneous values may show finer temporal and spatial variations than the ones provided by the model and their effects would need to be considered if this concept is brought to operational use.

- *Asm.7*: The atmosphere co-rotates and its wind behaves as predicted by the HWM07 model.

Figure 3B shows the sideslip angle caused by the atmospheric co-rotation and wind (using the HWM07 model) assuming that a spacecraft is aligned with its inertial velocity in a 10:30 LTAN circular Sun-synchronous orbit at different altitudes in moderate solar activity (ISO 14222, 2013).

### 4.3. Gas-Surface Interaction Model

In the orbital environment (>200 km in altitude) the residual atmosphere can no longer be considered as a continuum but, given its low density, needs to be considered as a rarefied-gas (Bird, 1994). The mean free path λ of an atmospheric gas particle is, in general, much greater than a representative spacecraft dimension (λ>100 m at 200 km altitude; Virgili-Llop, 2014). Consequently, it can be assumed that the interactions between gas particles (collisions) are very rare, and thus they can be safely neglected. Therefore, the Gas-Surface Interactions (GSI) completely dominate the interaction of the spacecraft with its surrounding gas.

- *Asm.8*: Gas-gas particle interactions are negligible.

The GSI are dependent on several gas and surface parameters. As these interactions occur at the molecular scale, molecular scale parameters are also relevant (e.g., lattice configuration and surface roughness among others). The high thermal velocity of the gas particles (~1 km/s at 350 km), due to the high temperature and low density of the gas, produces a flow that is not collimated. The non collimated flow leads to particles colliding with surfaces that would intuitively appear to be shadowed from the flow.

There are several GSI models (Bird, 1994) and in this study the Sentman model (Sentman, 1961) will be used as it is the *de facto* standard to compute spacecraft aerodynamic coefficients at low altitudes (Moe and Moe, 2005, 2010). A comprehensive description of the models used in spacecraft aerodynamics can be found elsewhere (Moe and Moe, 2010; Prieto et al., 2014).

- *Asm.9*: The lift and drag coefficients behave according to the Sentman model.

The Sentman model takes into account the thermal velocity distribution of the gas particles and assumes that all the incident gas particles that collide with a surface are adsorbed to be later diffusely reemitted. In the LEO range this seems to be true from the limited available orbital data (Gregory and Peters, 1987; Moe et al., 1998). The particles are then reemitted with partial thermal equilibrium with the spacecraft surface. The degree of thermal equilibrium is denoted by the energy accommodation coefficient σ_*a*_. In this model, the *C*_*d*_ and *C*_*l*_ can be written, following a notation similar to Sutton (2009) and Doornbos (2011), as in Equations (23, 25).

(23) $$\begin{array}{l} C_{d}=\left[\frac{P}{\sqrt{\pi }}+\gamma QZ+\frac{\gamma }{2}\frac{v_{\text{re}}}{V_{\infty }}\left(\gamma \sqrt{\pi }Z+P\right)\right] \end{array}$$

(24) $$\begin{array}{l} C_{D}=\frac{\int C_{d}dA}{A_{\text{ref}}} \end{array}$$

(25) $$\begin{array}{l} C_{l}=\left[lGZ+\frac{l}{2}\frac{v_{\text{re}}}{V_{\infty }}\left(\gamma \sqrt{\pi }Z+P\right)\right] \end{array}$$

(26) $$\begin{array}{l} C_{L}=\frac{\int C_{l}dA}{A_{\text{ref}}} \end{array}$$

(27) $$\begin{array}{l} \gamma =\text{cos }\left(\varphi \right) \end{array}$$

(28) $$\begin{array}{l} l=\text{sin }\left(\varphi \right) \end{array}$$

(29) $$\begin{array}{l} G=\frac{1}{2s^{2}} \end{array}$$

(30) $$\begin{array}{l} P=\frac{1}{s}e^{-\gamma^{2}s^{2}} \end{array}$$

(31) $$\begin{array}{l} Q=1+G \end{array}$$

(32) $$\begin{array}{l} Z=1+\text{erf}\left(\gamma s\right) \end{array}$$

The φ term denotes the angle between the flow and the surface local normal vector (0° when the surface is normal to the flow and 90° when it is parallel), *v*_re_ the most probable velocity of the reemitted gas particles, *V*_∞_ the relative bulk velocity between the spacecraft and the incident gas particles (the same one as in Equations 20–22), *A*_ref_ an arbitrary reference area (usually the cross section area of the spacecraft), *s* the ratio between *V*_∞_ and the most probable thermal velocity of the gas *v*_th_ ($s=\frac{V_{\infty }}{v_{\text{th}}}$), and erf (*x*) denotes the error function.

According to Koppenwallner (2009) the *v*_re_/*V*_∞_ ratio can be written as in Equation (33), with *R*_*g*_ denoting the gas constant and *T*_*w*_ the temperature of the surface (wall).

(33) $$\begin{array}{l} \frac{v_{\text{re}}}{V_{\infty }}=\sqrt{\frac{1}{2}\left[1+\sigma_{a}\left(\frac{4R_{g}T_{w}}{V_{\infty }^{2}}-1\right)\right]} \end{array}$$

In the VLEO range the atomic oxygen is one of the dominant species. These atomic oxygen gas molecules get adsorbed into the spacecraft surfaces masking the original surface properties. Having a surface covered with atomic oxygen rises the accommodation coefficient to a level between 0.8 and 1 (Moe and Moe, 2005). The spacecraft surface temperature will be assumed constant at *T*_*w*_ = 300K.

Note that the drag and lift coefficients are dependent on the atmospheric parameters through the *v*_re_/*V*_∞_ and *s* parameters. As the atmosphere has temporal and spatial variability (vertical but also horizontal) the force coefficients will in general be variable during an orbit. These changes in the force coefficients are small and can be safely ignored given that the variability of the atmosphere (changes in atmospheric density and relative flow direction and magnitude) is orders of magnitude larger. Additionally, although the Sentman model can provide the lift coefficient *C*_*L*_, it is, in general, an order of magnitude smaller than the drag coefficient *C*_*D*_ and thus it will be neglected in this study (Doornbos, 2011).

- *Asm.10*: The changes of *v*_re_/*V*_∞_ and *s* during an orbit are negligible when compared to the atmospheric density variability.

### 4.4. Aerodynamic Properties of a Sphere

Equation (34) can be used to compute the drag coefficient *C*_*D*_ of a sphere. The reference area is set as the cross section area of the sphere $A_{\text{ref}}=\pi R^{2}$. In Equation (34), θ_*sc*_ and ϕ_*sc*_ are the azimuthal and polar spherical coordinate angles.

(34) $$C_{D}=\frac{\int C_{d}\left(\varphi \right)dA}{\pi R^{2}}=\frac{\int_{0}^{\pi }\int_{0}^{2\pi }C_{d}\left(\theta_{sc},\psi_{sc}\right)\text{ sin }\phi_{sc}d\theta_{sc}d\phi_{sc}}{\pi }$$

Equation (35) computes the angle between the flow and the local normal vector φ (required by the Sentman model) using the polar spherical coordinate angles.

(35) $$\begin{array}{l} \text{cos }\varphi =\text{cos }\left(\pi /2-\phi_{sc}\right)\text{cos }\theta_{sc} \end{array}$$

**Figure 4:**
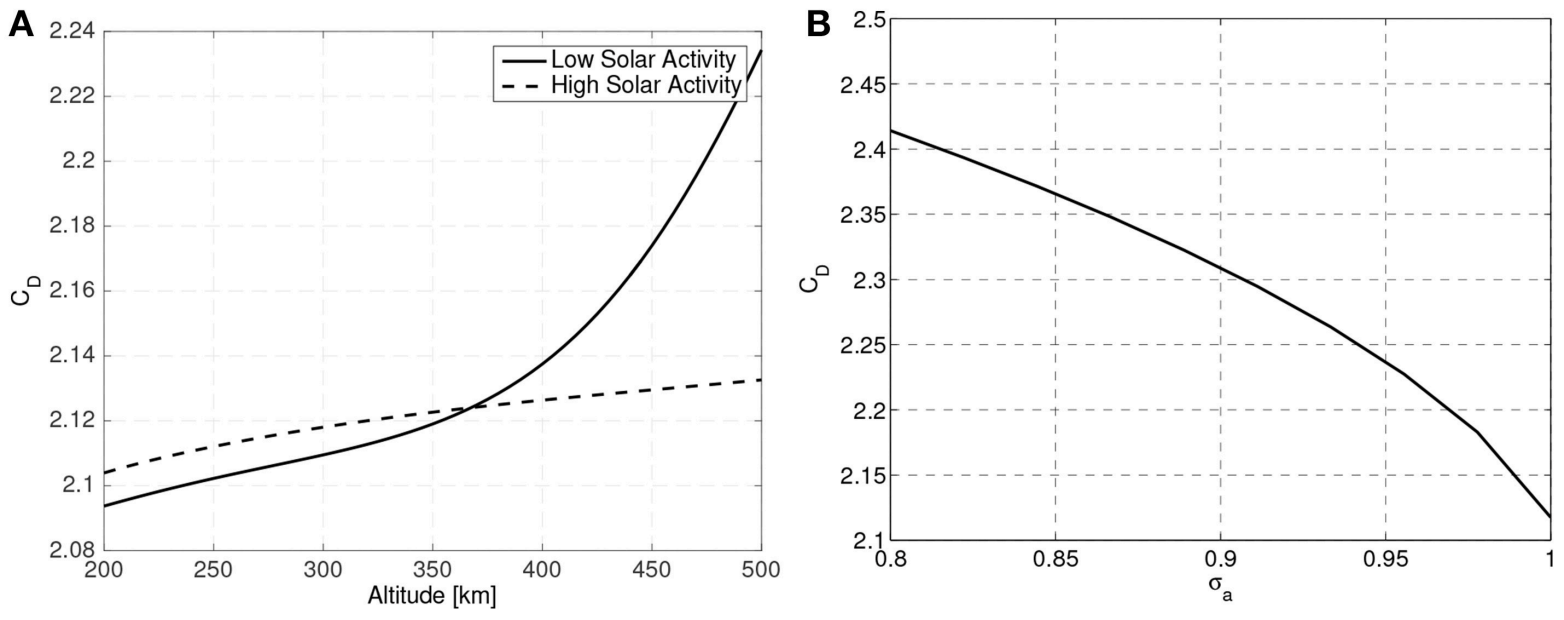
Variation of a sphere drag coefficient with altitude, solar activity **(A)** and energy accommodation coefficient **(B)**.

Due to the sphere's symmetry, the drag coefficient is constant regardless of the orientation of the sphere with respect to the flow (greatly simplifying the analysis). By using Equation (34) a sphere's drag coefficient is found to be around *C*_*D*_ ≈ 2.1. Figure 4 clearly shows how the drag coefficient of a sphere changes with altitude, solar activity and energy accommodation coefficient, making this magnitude variable and uncertain.

The orientation of the body reference frame *B*_0_ with respect to the orbital frame will be denoted by the common roll ϕ, pitch θ and yaw ψ Euler angles. When roll, pitch and yaw are 0 the body frame is aligned with the orbit frame. The relative flow direction will be defined with its own reference frame where the flow direction will be in $-{\hat{i}}_{\text{flow}}$ axis. The orientation of this flow reference frame will be denoted by a flow pitch θ_flow_ and flow yaw ψ_flow_.

Let ***R***_*OB*_ denote the rotation matrix from body to orbital and ***R***_*OF*_ the rotation matrix from flow to orbital and thus $R_{BF}=R_{OB}^{T}R_{OF}$ is the rotation from the flow to the body reference frame. With these definitions the aerodynamic force in body axes is defined by Equation (36), with *D* denoting the aerodynamic drag.

(36) $$\begin{array}{l} F_{\text{aero}}=R_{BF}\left[\begin{array}{c} -D\\ 0\\ 0 \end{array}\right] \end{array}$$

**Figure 5:**
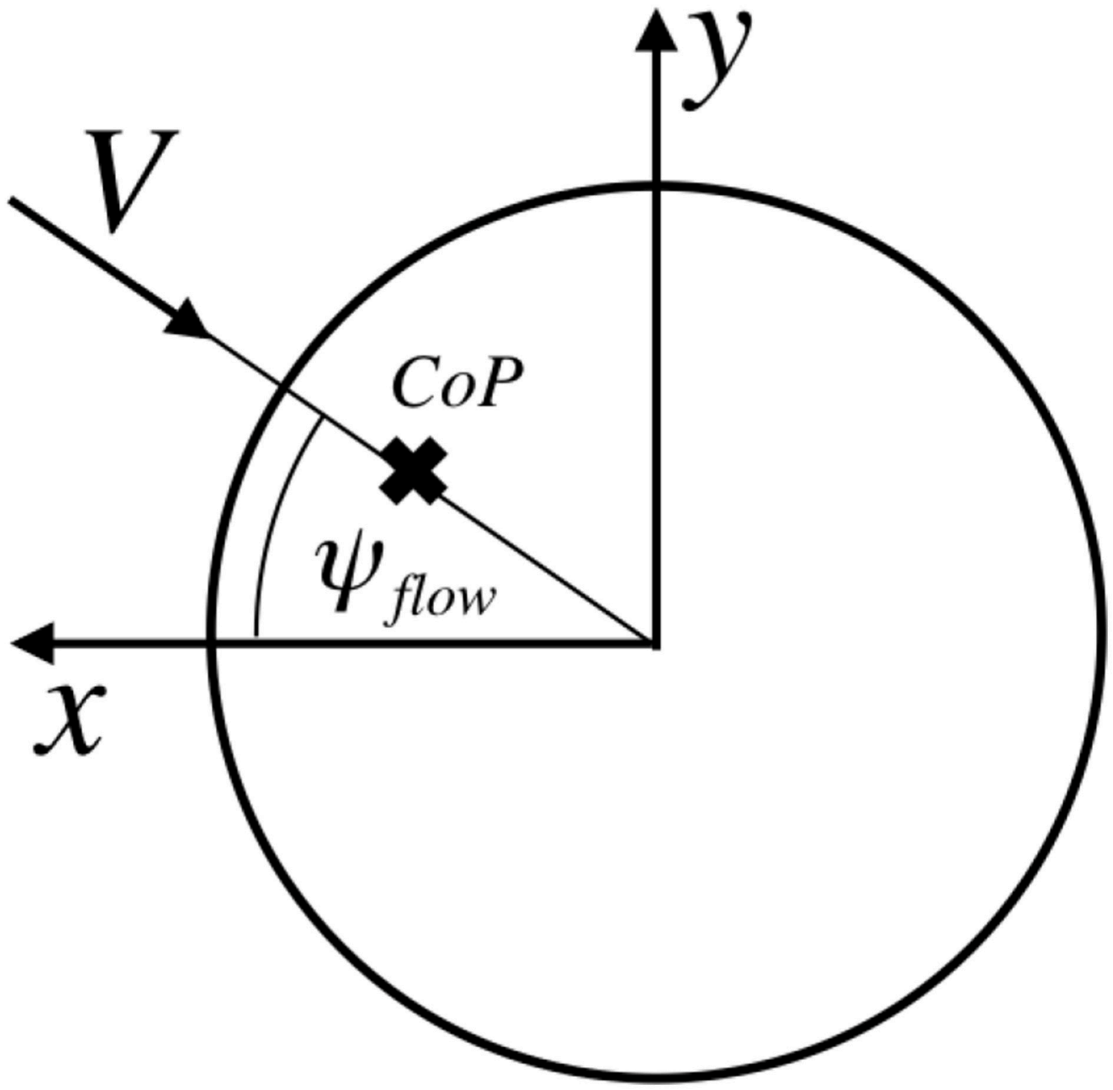
Location of the sphere's Center-of-Pressure.

The CoP of the sphere is aligned with the direction of the flow ${\hat{i}}_{\text{flow}}$ (see Figure 5). The location of the center of pressure from the sphere's center can be computed using (Equation 37). Again this magnitude is slightly dependent on the altitude but it will be assumed as constant (it will eventually be shown that the location of the real CoP of the sphere is not relevant).

(37) $$\begin{array}{l} \frac{d_{\text{CoP}}}{R}=\frac{\int C_{d}\left(\varphi \right)xdA}{\pi R^{2}C_{D}}\\ \;=\frac{\int_{0}^{\pi }\int_{0}^{2\pi }C_{d}\left(\theta_{sc},\phi_{sc}\right)\sin^{2}\phi_{sc}\text{cos }\theta_{sc}d\theta_{sc}d\phi_{sc}}{\pi C_{D}}\approx 0.66 \end{array}$$

The location of the spacecraft CoP in body axes *p*_CoP_ can then be written as in Equation (38).

(38) $$\begin{array}{l} p_{\text{CoP}}=\left[\begin{array}{c} -d_{CoM_{0}}\\ 0\\ 0 \end{array}\right]+R_{BF}\left[\begin{array}{c} d_{CoP}\\ 0\\ 0 \end{array}\right] \end{array}$$

Due to the sphere's symmetry, the relative flow ${\hat{i}}_{\text{flow}}$, the CoP, and the sphere's geometric center are aligned. Therefore, there is no torque with respect to the sphere's geometric center. The aerodynamic torque with respect the host vehicle CoM is then only a function of *d*_Co_M__0__ as shown in Equation (39). It can then be assumed that torque-wise, the effective CoP is located at the sphere's geometric center.

(39) $$\begin{array}{l} \tau_{\text{aero}}=p_{\text{CoP}}\times F_{\text{aero}}=\left[\begin{array}{c} -d_{\text{Co}{\text{M}}_{0}}\\ 0\\ 0 \end{array}\right]\times F_{\text{aero}} \end{array}$$

Although in this work a spherically shaped spacecraft has been used, an analogous analysis can be conducted for spacecraft with more complex shapes.

It may be useful when devising the controllers to simplify these aerodynamic force and torque equations. If the control method fulfills its goal the spacecraft attitude will be in close vicinity of its target attitude ϕ ≈ 0, θ ≈ 0, ψ ≈ 0 (small angles approximation). Additionally, the atmospheric co-rotation and wind do not cause the relative flow to have large deviations with respect to the inertial velocities (see Figure 3B) making θ_flow_ and ψ_flow_ also small. Under these assumptions, the Euler angles of the spacecraft with respect to the flow (rotation represented by *R*_*BF*_) can be approximated using ϕ′ = −ϕ, $\theta^{\prime }=\theta_{\text{flow}}-\theta$ and $\psi^{\prime }=\psi_{\text{flow}}-\psi$ (which will also be small angles) and the aerodynamic forces in body axes can be subsequently approximated by Equation (40).

(40) $$\begin{array}{l} F_{\text{aero}}\approx -D\left[\begin{array}{c} \text{cos }\left(\theta_{\text{flow}}-\theta \right)\text{cos }\left(\psi_{\text{flow}}-\psi \right)\\ \text{cos }\left(\theta_{\text{flow}}-\theta \right)\text{sin }\left(\psi_{\text{flow}}-\psi \right)\\ -\text{sin }\left(\theta_{\text{flow}}-\theta \right)\text{cos }\left(\psi_{\text{flow}}-\psi \right) \end{array}\right]\approx D\left[\begin{array}{c} -1\\ -\psi^{\prime }\\ \theta^{\prime } \end{array}\right] \end{array}$$

A simplified expression for the aerodynamic torque can also be obtained using the same small angle approximation as shown in Equation (41).

(41) $$\begin{array}{l} \tau_{\text{aero}}\approx D\left[\begin{array}{c} 0\\ \theta^{\prime }d_{\text{Co}{\text{M}}_{0}}\\ \psi^{\prime }d_{\text{Co}{\text{M}}_{0}} \end{array}\right] \end{array}$$

From Equation (41) it can be clearly seen that the equilibrium attitude is that attitude where the flow, the host vehicle CoM, and the sphere's geometric center are aligned (θ′ = ψ′ = 0). When there is a misalignment of this equilibrium attitude, the aerodynamic torques will provide a restoring torque if the sphere center is behind the host vehicle CoM *d*_*M*_*p*__ > 0, making the spacecraft oscillate around this equilibrium point (marginally stable). If the host vehicle CoM is leading the center of the sphere *d*_*M*_*p*__ < 0 the system is unstable.

In a marginally stable configuration *d*_*M*_*P*__ > 0 the natural frequency of the oscillation can be approximated (using the small angles approximation) with Equation (42).

(42) $$\begin{array}{l} \omega_{n}=\sqrt{\frac{Dd_{\text{Co}{\text{M}}_{0}}}{I}} \end{array}$$

**Figure 6:**
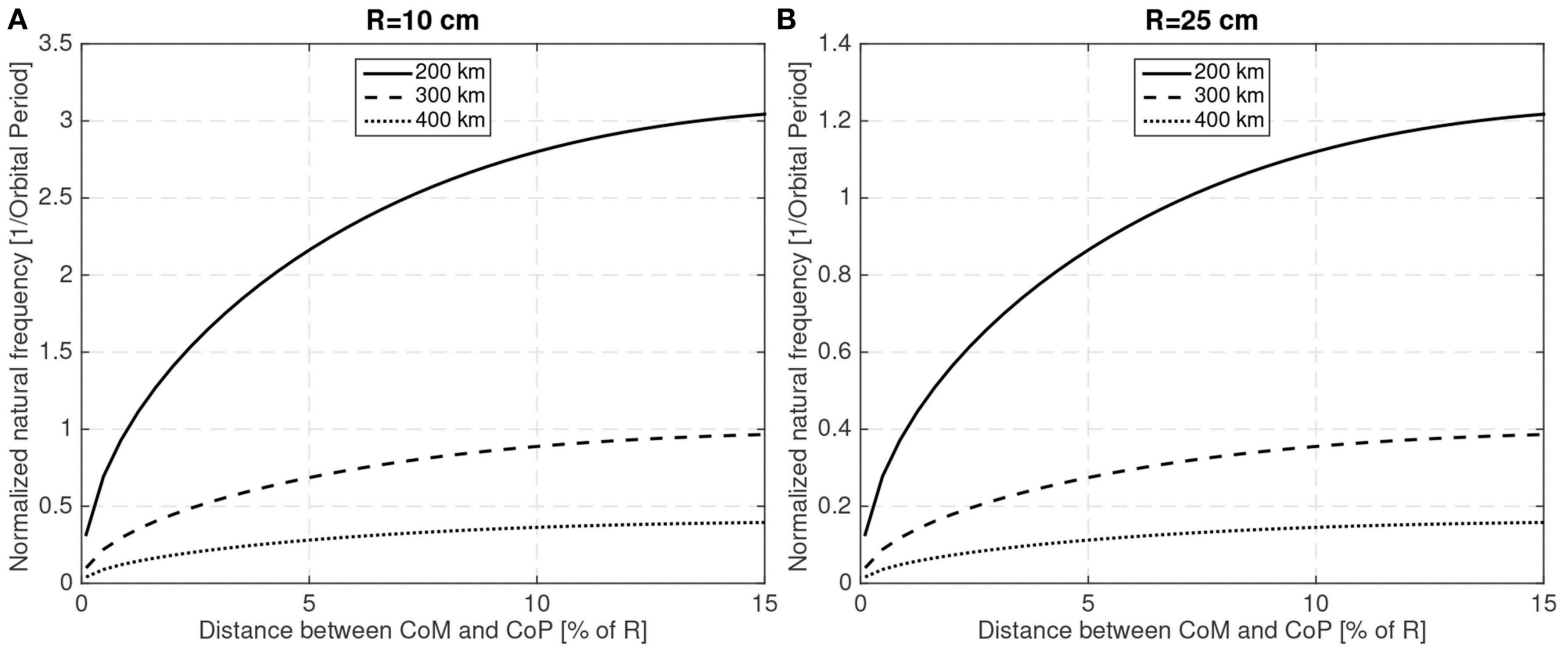
Natural frequency (normalized with the orbit period) for different CoM and altitudes for *R* = 10 cm **(A)** and *R* = 25 cm **(B)**.

The natural frequency will, in general, be small and thus it can be normalized with the orbital mean motion to make it easier to read. Figure 6 shows the natural frequency (normalized with the orbit period) for two different sphere sizes (*R* = 10 cm and *R* = 25 cm) with different CoM to CoP distances, at different altitudes and using the numerical parameters shown in Table 1.

**Table 1:** Numerical parameters.

**Parameter**	**Value**
κ	0.1
ρ_*s*_	500 kg m^2^
*C*_*D*_	2.2
σ_*C*_*D*__	0.22 (10 % of the nominal *C*_*D*_ = 2.2)
Solar activity indices	Moderate activity as in ISO 14222 (2013).

As the magnitude of the aerodynamic force is proportional to the area ~ *R*^2^, larger spacecraft with equivalent natural frequencies will exhibit smaller perturbations as will have larger inertias ~ *R*^5^. Another expected result is that as the altitude increases and the aerodynamic disturbance weakens, the natural frequency also decreases. Thus, it is readily apparent that the aerodynamic disturbances will be more important for small spacecraft at low altitudes. The simulations that have been conducted have then been focused on small spacecraft examples.

It is important to note that by definition, the host vehicle CoM is displaced with respect to the sphere center only along the *B*_0_ $\hat{i}$ direction. This condition has been imposed to simplify the analysis but it is expected to be met by VLEO spacecraft. In a generic case, the CoM can be displaced in any direction and then a secular aerodynamic torque will appear when the spacecraft is at the target attitude (ignoring the direction variability of the relative flow direction). Therefore, it is highly desirable to have the CoM and effective CoP (center of the sphere) aligned with the *B*_0_ $\hat{i}$ axis in order to avoid these secular torques. As it is expected that VLEO spacecraft designers will take this issue into consideration it can be safely assumed that the CoM and CoP, for the target attitude, will be reasonably aligned. Any residual misalignment can be corrected by a bias in the position of the shifting masses (resulting in a reduction of their shifting range and control authority) and thus the initial assumption of host vehicle CoM displaced only along the *B*_0_ $\hat{i}$ direction can be recovered (without loss of generality).

The mission of the shifting masses is then to stabilize the spacecraft in the presence of these aerodynamic attitude disturbance eliminating the need to use other actuators for this purpose (thus potentially delaying saturation and saving power and mass).

The assumption that the host vehicle CoM is displaced with respect to the sphere center roughly along the *B*_0_ $\hat{i}$ direction represents one of the limitations of the proposed method. The shifting masses are only able to reject the aerodynamic disturbances for the limited set of attitudes where this assumptions holds. For arbitrary attitudes, the aerodynamic torques may be too strong to be compensated by the shifting masses. However, in the context of VLEO, these strong secular torques may be a burden for other attitude control methods as well (e.g., rapidly saturating reaction wheels) and thus holding attitudes far from the aerodynamic equilibrium points is an intrinsic challenge for spacecraft operating in VLEO.

## 5. Rejection Capabilities Under a Reduced Model With One Rotational Degree-of-Freedom

A potential application of the shifting masses is disturbance rejection. For simplicity, it is worth to start the rejection capability analysis with a reduced model that only considers one rotational degree-of-freedom. This analysis provides insight into the rejection capabilities and the shifting mass system requirements with respect to the system's parameters. It is also of particular interest to explore how the spacecraft size and operating altitude drive the required shifting mass and range in order to meet pre-specified performance requirements.

The yaw ψ rotation has been selected for this one-dimensional analysis as the co-rotation and predominant wind act on this particular axis. Additionally, a single shifting point mass will be used and the controller will be based on linearized dynamics. The goal of the shifting mass is then to stabilize the spacecraft around ψ = 0 and reject the disturbance induced by ψ_flow_.

- *Reduced Model Controller Asm.1*: Single rotational degree-of-freedom (yaw axis).
- *Reduced Model Controller Asm.2*: Shifting mass is a point mass.
- *Reduced Model Controller Asm.3*: The mass and inertia properties of the host vehicle and of the shifting mass are known.
- *Reduced Model Controller Asm.4*: The relative position, velocity and acceleration of the shifting mass are known.

Under these assumptions, the equation of motion in Equation (19) can be further simplified, yielding Equation (43). The position, velocity and acceleration of the unique shifting mass with respect to the body axes is denoted by *x, y* and the shifting mass velocity with respect to the body reference frame *B*_0_ by ẋ′, ẏ′.

(43) $$\begin{array}{r} \left[J_{z}+\mu \left(x^{2}+y^{2}\right)\right]\ddot{\psi }+\mu \left[2\left(x{ẋ}^{\prime }+y{ẏ}^{\prime }\right)\dot{\psi }+x{ý}^{\prime }-y{\ddot{y}}^{\prime }\right]\\ =\tau_{z}+\frac{\mu }{M_{0}}\left[F_{x}y-F_{y}x\right] \end{array}$$

The aerodynamic disturbances have low frequencies (similar to the orbit frequency) and so it is expected that the motion of the shifting mass will be also slow (small velocities and accelerations), thus limiting the dynamic effects of the shifting mass. Additionally, as the shifting mass *m* is small compared to the host vehicle mass μ ≪ 0, the dynamic effects of the shifting mass will be further reduced and they can therefore be safely neglected during the controller design.

- *Reduced Model Controller Asm.5*: Shifting mass velocities and accelerations have negligible effects on the dynamics.

As the shifting range is also small the change on the system's inertia is also small and thus the system's inertia will be considered as constant (using the initial shifting masses position *x*_0_ and *y*_0_) during the controller design. These assumptions further simplify the equations of motion to Equation (44). It also has to be noted that only aerodynamic forces and torques will be considered.

(44) $$\begin{array}{l} \left[J_{z}+\mu \left(x_{0}^{2}+y_{0}^{2}\right)\right]\ddot{\psi }=\tau_{z}+\frac{\mu }{M_{0}}\left[F_{x}y-F_{y}x\right] \end{array}$$

- *Reduced Model Controller Asm.6*: Constant system inertia.

The shifting masses modulate ${\hat{\tau }}_{\text{aero}}$ by actively changing the location of the system CoM. Using the aerodynamic properties of a sphere and using the small angles approximation, Equation (45) can be obtained.

(45) $$\begin{array}{l} \left[J_{z}+\mu \left(x_{0}^{2}+y_{0}^{2}\right)\right]\ddot{\psi }=D\left(\psi^{\prime }d_{\text{Co}{\text{M}}_{0}}+\frac{\mu }{M_{0}}\left[-y+\psi^{\prime }x\right]\right) \end{array}$$

- *Reduced Model Controller Asm.7*: The system remains at all times close to its target attitude (small angles approximation).

It is immediately clear from Equation (45) that to generate a control torque it is much more effective for the mass to move perpendicular to the relative flow (in this case *y*) than parallel to it (along *x*). So in order to limit the system complexity, it will be assumed that the shifting mass only moves in *y* (perpendicular to the flow if ψ′ is small). Shifting the mass only along one direction reduces the volume and the complexity of the shifting mass system while maximizing its effectiveness. It is understood that (Equation 45) has been simplified for small angles and thus shifting the mass along *y* will only be perpendicular to the flow direction only for ψ = ψ_*flow*_ = 0, if there is a large misalignment the *y* shifting mass will start to loose efficacy.

- *Reduced Model Controller Asm.8*: The shifting mass only moves along the $\hat{j}$ axis.

Another important consideration that it is apparent from Equation (45) is that the maximum torque provided by the shifting mass is $\tau_{\text{max}}=\pm D\frac{m}{M_{0}+m}y_{\text{max}}$. It is clear that the mass of the shifting mass and the available shifting range are the two variables at the designer disposal to regulate the control authority of the system.

The atmospheric density and the magnitude and direction of the flow are inherently unknown to the controller. An estimated density, purely based on the altitude (no horizontal variability) will be used by the controller. Additionally, the controller will assume that the incident flow matches the inertial velocity magnitude and direction.

- *Reduced Model Controller Asm.9*: Constant atmospheric density.
- *Reduced Model Controller Asm.10*: Relative flow velocity matches the spacecraft's inertial velocity.

Under these conditions, the system equations can be written as Equation (46) which corresponds to the transfer function written in Equation (47). This represents a simple second order Single Input Single Output system and a Proportional-Integral-Derivative (PID) controller can be easily designed and implemented to reject the aerodynamic disturbances while keeping the spacecraft stable at ψ = 0.

(46) $$\begin{array}{l} \left[J_{z}+\mu \left(x_{0}^{2}+y_{0}^{2}\right)\right]\ddot{\psi }=D\left(-\psi \left[\frac{\mu }{M_{0}}x_{0}+d_{\text{Co}{\text{M}}_{0}}\right]-\frac{\mu }{M_{0}}y\right) \end{array}$$

(47) $$\begin{array}{l} T\left(s\right)=\frac{\psi \left(s\right)}{y\left(s\right)}=\frac{b}{J^{\prime }s^{2}\;+\;k} \end{array}$$

(48) $$\begin{array}{l} J^{\prime }=J_{z}+\mu \left(x_{0}^{2}+y_{0}^{2}\right) \end{array}$$

(49) $$\begin{array}{l} k=D\left(\frac{\mu }{M_{0}}x_{0}+d_{\text{Co}{\text{M}}_{0}}\right) \end{array}$$

(50) $$\begin{array}{l} b=-D\frac{\mu }{M_{0}} \end{array}$$

This controller also carries the underlying assumption that the shifting mass can instantaneously move, without lag, from one position to another one. This will be relaxed in subsequent sections.

- *Reduced Model Controller Asm.11*: Shifting mass movement has infinite bandwidth.

To explore the design space and the system response it will be assumed that the PID controller is tuned so that the closed loop system has a specific bandwidth and phase margin. In these fixed controller conditions a Montecarlo simulation can be performed to extract the required shifting mass range for a given spacecraft size and the uncertain aerodynamic properties and environmental conditions.

Although the controller is build upon a linearized model (see all *Reduced Model Controller Asm*.), the numerical simulations use the full dynamic equations and the high-fidelity environment models (only using the generic *Asm*.). To emulate the uncertainty on the aerodynamic properties, the actual drag coefficient used in the numerical simulation differs from the one used to design the controller. Although the CoP is considered known, given that a spherical shape is used, the uncertainty on the drag coefficient can also be used to emulate an uncertainty in the CoP position. Sun-synchronous circular orbits with 10:30 am mean LTAN have been used.

The Montecarlo simulations are initialized with ideal stable attitudes ψ = 0 and $\dot{\psi }=0$ and thus emulate steady state conditions. Each Montecarlo run simulates 4 consecutive orbits and 100 simulations are used to extract the statistics (with error bars denoting the 95% confidence interval).

**Figure 7:**
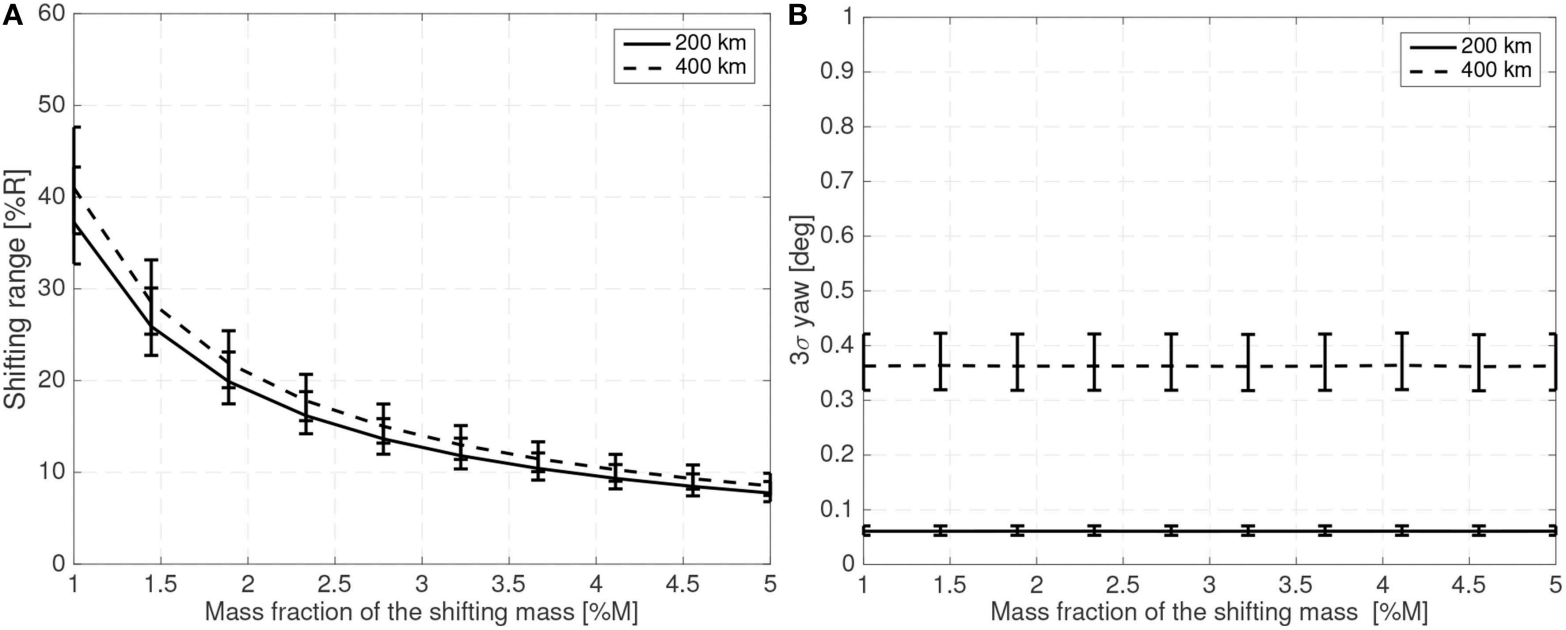
Required 3σ shifting range **(A)** and 3σ attitude error **(B)** with respect to the shifting mass fraction for a 10 cm radius spherical spacecraft.

**Figure 8:**
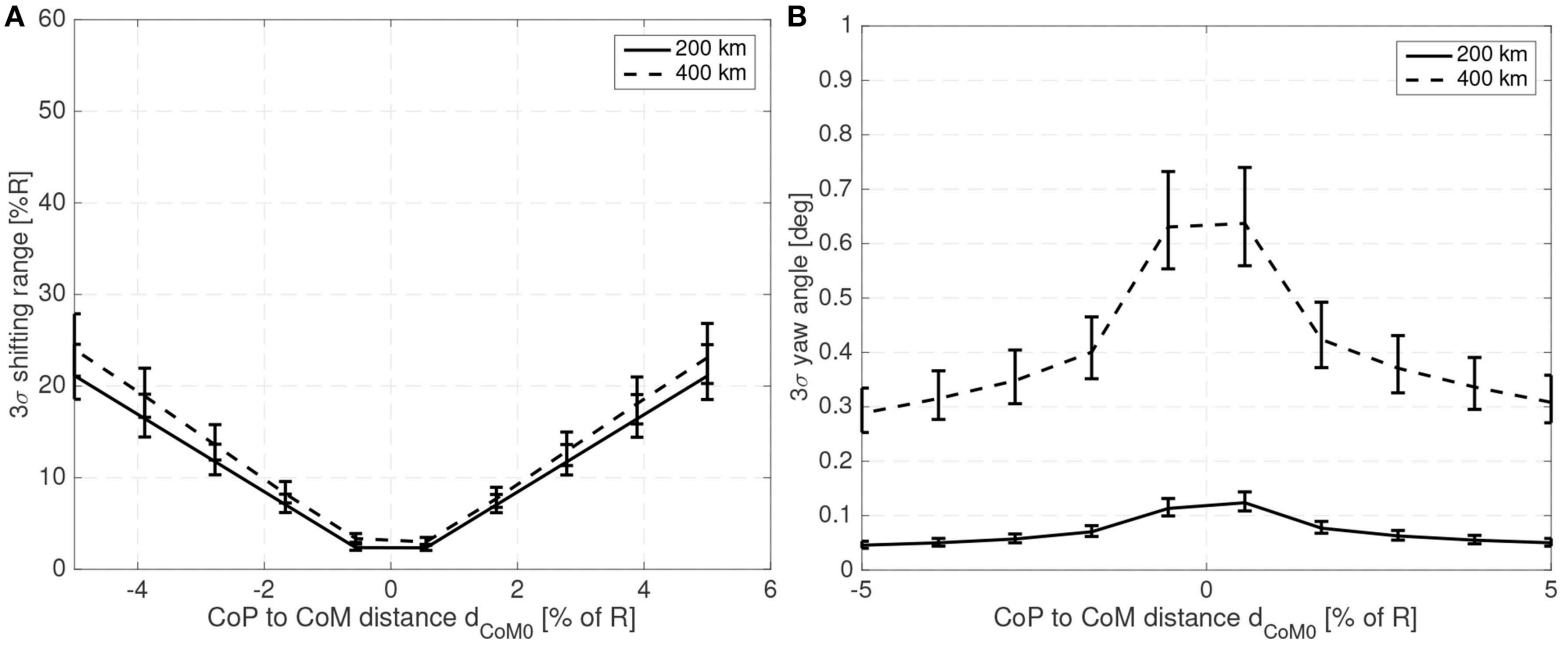
Required 3σ shifting range **(A)** and 3σ attitude error **(B)** with respect to the CoP to CoM distance *d*_Co_M__0__ for a 10 cm radius spherical spacecraft.

Figure 7 shows the maximum shifting range and attitude error (3σ values) for a 10 cm radius spherical satellite for different mass fractions of the shifting mass *m*/*M*_0_ and for a CoM leading the CoP by 3% of the sphere radius *R*. Figure 8 shows how the required shifting range and attitude error change for different CoP to CoM distances *d*_Co_M__0__ and with a fixed shifting mass fraction kept at 3% of the host vehicle mass *M*_0_.

The system bandwidth in the controllers used to generate (Figures 7,8) has been kept at four times the natural frequency of the system 4ω_*n*_ and the phase margin set to 30 deg. This allows a comparison of the system performance even if the altitude or spacecraft size are changed.

For the 10 cm radius case it is quite clear that the proposed method is able to reject the aerodynamic disturbances and maintain a reasonably stable attitude (with respect to 10 cm sized spacecraft standards Polat et al., 2016) with mass fraction and shifting range requirements compatible with the spacecraft mass and dimension constrains (considering that realistic uncertainty in the parameters has been taken into account). Note how the proposed method is able to stabilize the spacecraft even if the CoM is behind the CoP (unstable configuration). As expected the required mass fraction and required shifting range decrease as the CoP gets closer to the CoM. It is also worth pointing that the unstable configuration *d*_*p*_ < 0 requires higher shifting range than their stable counterparts.

**Figure 9:**
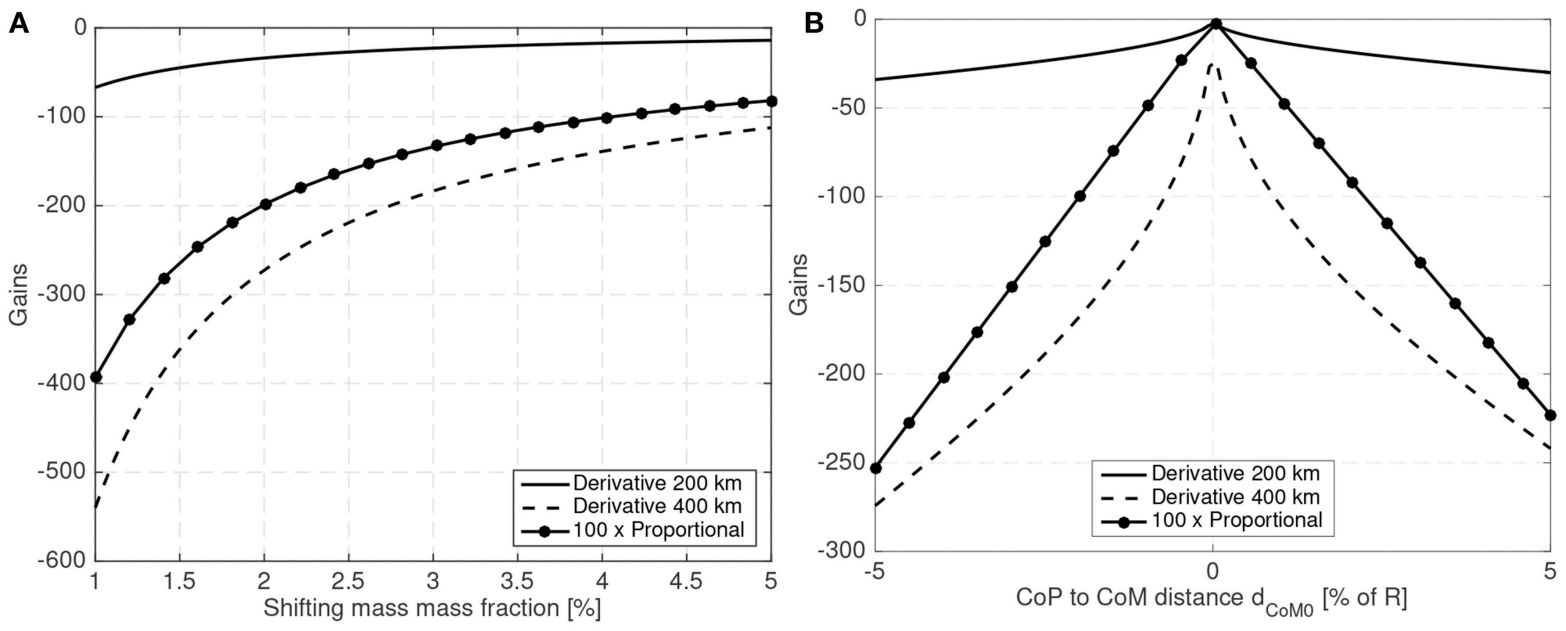
PID gains **(A)** for different mass fractions and **(B)** for different CoP to CoM distances *d*_Co_M__0__ for a 10 cm radius spherical spacecraft.

The attitude error, which is constant in Figure 7 due to the constant bandwidth employed, can be decreased if the bandwidth of the close looped system is increased. The required shifting range can be decreased by decreasing the phase margin. But both measures have limits. By decreasing the phase margin the controller is less robust and increasing the bandwidth increases the gains which imposes more strict requirements on the sensors and actuators. The PID gains for the 10 cm radius case are shown in Figure 9 when the angular and angular velocity errors are provided in rad and rad/s. As expected, the gains increase as the system becomes more difficult to control, that is with increasing *d*_Co_M__0__ or reducing mass fraction of the shifting mass. It is important to note that the high derivative gain may impose certain requirements on the attitude velocity estimates that the attitude determination subsystem needs to provide to the controller (specially in terms of noise levels).

**Figure 10:**
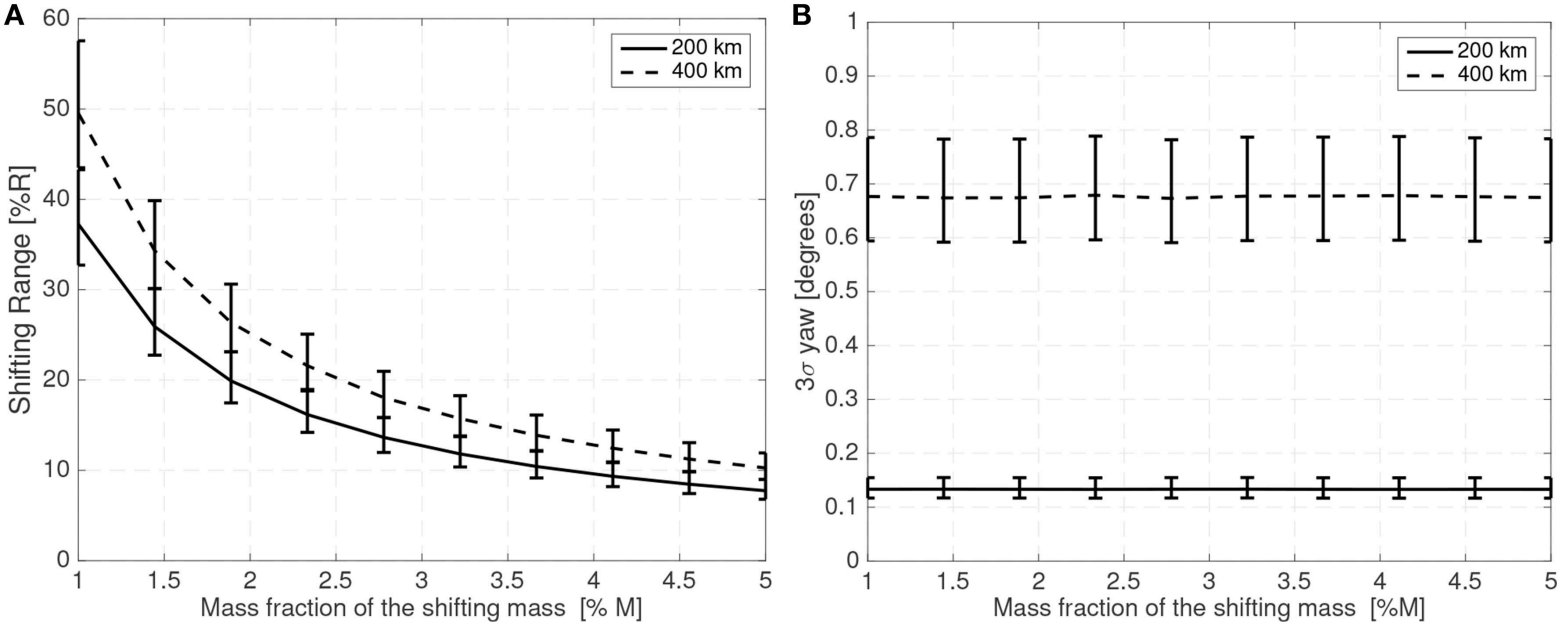
Required shifting range **(A)** and attitude error **(B)** with respect to the shifting mass fraction for a 25 cm radius spherical spacecraft.

**Figure 11:**
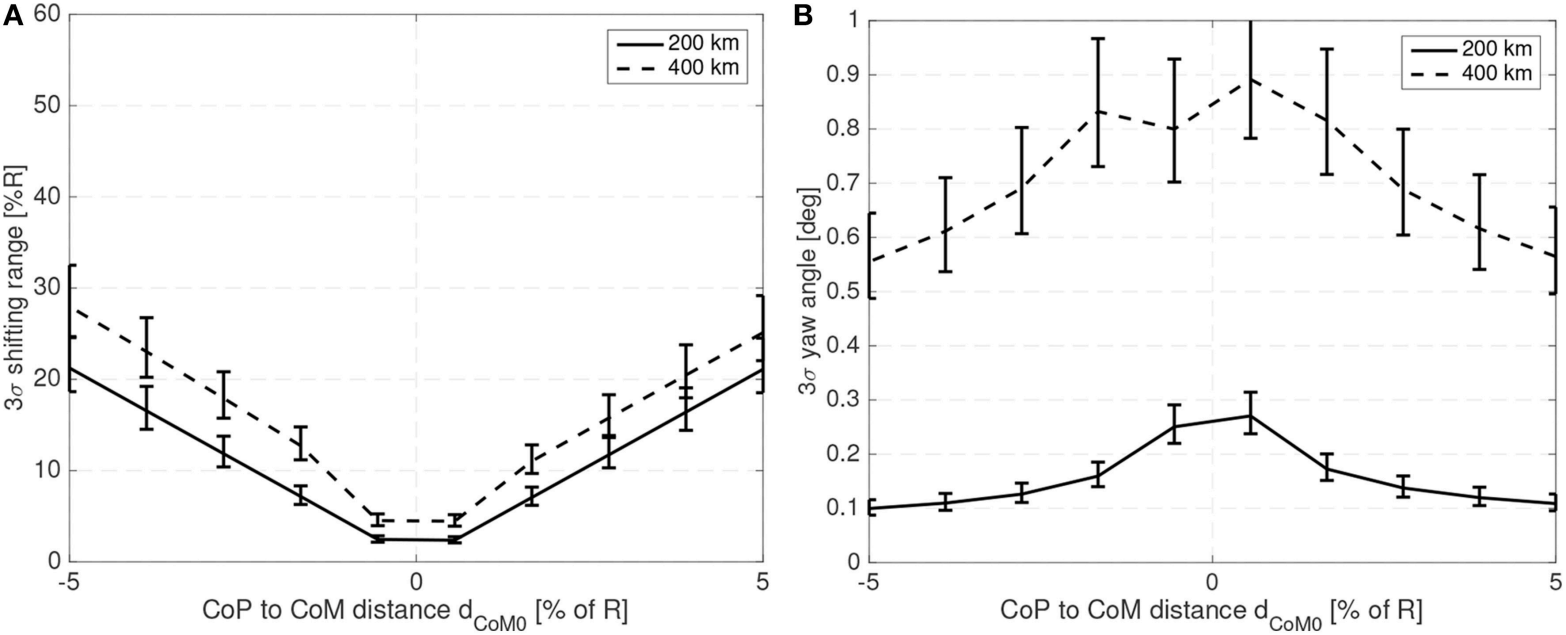
Required shifting range **(A)** and attitude error **(B)** with respect to the CoP to CoM distance for a 25 cm radius spherical spacecraft.

It is also interesting to note that the relative shifting range increases slightly with altitude or spacecraft size as a relative larger shifting is required to generate the same acceleration at higher altitude or for spacecraft with larger inertias. As in this analysis the controller bandwidth is kept constant relative to the natural frequency, larger spacecraft display larger attitude errors (as their natural frequency is significantly lower). For a 25 cm radius spherical satellite the attitude error and required shifting range for different altitudes, mass fractions, and CoP to CoM distances is shown in Figures 10, 11.

Lower natural frequencies do reduce the proportional and derivative gains and thus spacecraft of bigger size or operating at higher altitudes have a wider margin to increase their controller bandwidth and reduce the attitude error whilst maintaining reasonable gains.

The tuning employed in this examples appears to give satisfactory results with the selected parameters and uncertainties. These examples illustrate the general trends and provide performance estimates that can be later used as initial guesses.

## 6. Three Rotational Degrees-Of-Freedom Case

The previous analysis has been conducted using a reduced model and only considering a single rotational degree-of-freedom. That analysis is useful as provides generic results and shows the trends when the different parameters are varied. In this section two simple controllers for the three rotational degree-of-freedom case will be presented to demonstrate the applicability of the proposed method to a multidimensional case.

### 6.1. Shifting Masses Driver

For the PID controller it was assumed that the shifting masses motion had infinite bandwidth. As the movement of the masses was slow that did not pose any problem. In reality the controller will specify the position of shifting masses and then the masses will need to move to those positions using a limited acceleration and velocity. For the three rotational degrees-of-freedom controllers an underlying PID controller will control the motion of the shifting masses to their desired location.

Although the underlying shifting masses actuators may be capable of rapid motion and aggressive acceleration, these magnitudes may need to be bounded in order to limit the dynamic effects of the shifting masses motion (which are currently ignored by the controllers and thus could potentially degrade the controller's performance). This is specially relevant when the shifting masses controller may request abrupt shifting masses position changes (e.g., when alternating saturation positions are requested).

Equation (43) can be used to select these velocity and acceleration limits. For example these limits can be selected so that the effects due to the shifting masses velocity or acceleration is no greater than a certain fraction η of the aerodynamic torque created by that shifting mass, as shown in Equations (51, 52).

(51) $$\begin{array}{l} {ẏ}_{\text{max}}^{\prime }<\eta \frac{D}{2{\dot{\psi }}_{\text{max}}M_{0}} \end{array}$$

(52) $$\begin{array}{l} {\overset{˙˙}{y}}_{\text{max}}^{\prime }<\eta \frac{Dy_{\text{max}}}{M_{0}x_{0}} \end{array}$$

These proposed limits are mainly dominated by the host vehicle mass *M*_0_ and by the atmospheric density (through the drag *D*). As the host vehicle mass is proportional to the radius of the spacecraft these limits can also be set with respect of the vehicle size and in this analysis a simple shifting mass acceleration and velocity limit of *R*/10 m/s^2^ and *R* m/s respectively has been employed.

Another option which would regulate itself would be to include the dynamic effects of the shifting masses motions into the controller. The controller would then avoid sudden accelerations or try to compensate the dynamic effects caused by the shifting mass motion.

### 6.2. Linear Quadratic Regulator Approach

Given the good performance of the PID controller on a one rotational degree-of-freedom case, it seems that a Linear Quadratic Regulator (LQR) based controller may also have a good performance for an attitude hold scenario (steady state) in a three rotational degrees-of-freedom case. When the system is linearized around the equilibrium point all the non-linearities from the presence of the shifting masses disappear or are neglected as it is assumed that the shifting masses move slowly and their mass fractions are small. When building this controller many of the assumptions made for the PID one rotational degree-of-freedom controller are carried over.

- *LQR Controller Asm.1*: Linearized dynamics.
- *LQR Controller Asm.2*: Shifting masses are point masses.
- *LQR Controller Asm.3*: The mass and inertia properties of the host vehicle and of the shifting masses are known.
- *LQR Controller Asm.4*: The relative position, velocity and accelerations of the shifting masses are known.
- *LQR Controller Asm.5*: Shifting masses velocities and accelerations have negligible effects on the dynamics.
- *LQR Controller Asm.6*: Constant system inertia.
- *LQR Controller Asm.7*: The system remains at all times close to its target attitude (small angles approximation).
- *LQR Controller Asm.8*: Constant atmospheric density.
- *LQR Controller Asm.9*: Relative flow velocity matches the spacecraft's inertial velocity.

When the spacecraft is in the vicinity of its equilibrium point the masses that shift perpendicular to the relative flow provide the maximum efficacy. As the goal is to keep the spacecraft stable then using only two shifting masses (with mass *m*_1_ and *m*_2_), respectively moving along the *B*_0_ pitch $\hat{j}$ and yaw $\hat{k}$ axes, maximizes the available torque while minimizing the system's complexity and the required volume.

- *LQR Controller Asm.10*: Two masses moving along the $\hat{j}$ and $\hat{k}$ axes.

As the shifting masses are unable to provide any control torque parallel to the flow then an additional third actuator is required. In this analysis an ideal actuator acting on roll *î* providing τ_roll_ has been assumed.

- *LQR Controller Asm.11*. Ideal roll actuator to augment the otherwise underactuated system.

In the typical case where the system is in the vicinity of the equilibrium point, this configuration and the LQR type controller is well suited. For a detumbling case, where the system does not remain in the linear region, an LQR controller may be less efficient than other non-linear controllers. It is also worth mentioning that, as the aerodynamic force is the dominant perturbation and only acts perpendicular to the flow direction (nominally along pitch and yaw), it is expected that the roll actuator will not be required to provide significant torques when the system is in the stable around the target equilibrium attitude.

**Figure 12:**
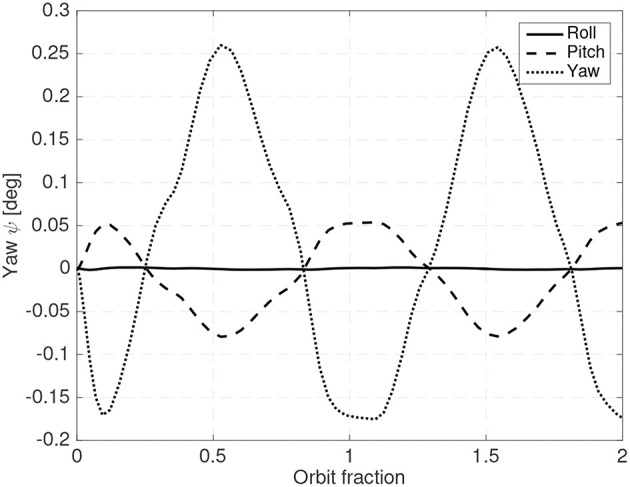
Roll, pitch and yaw using the LQR controller.

**Figure 13:**
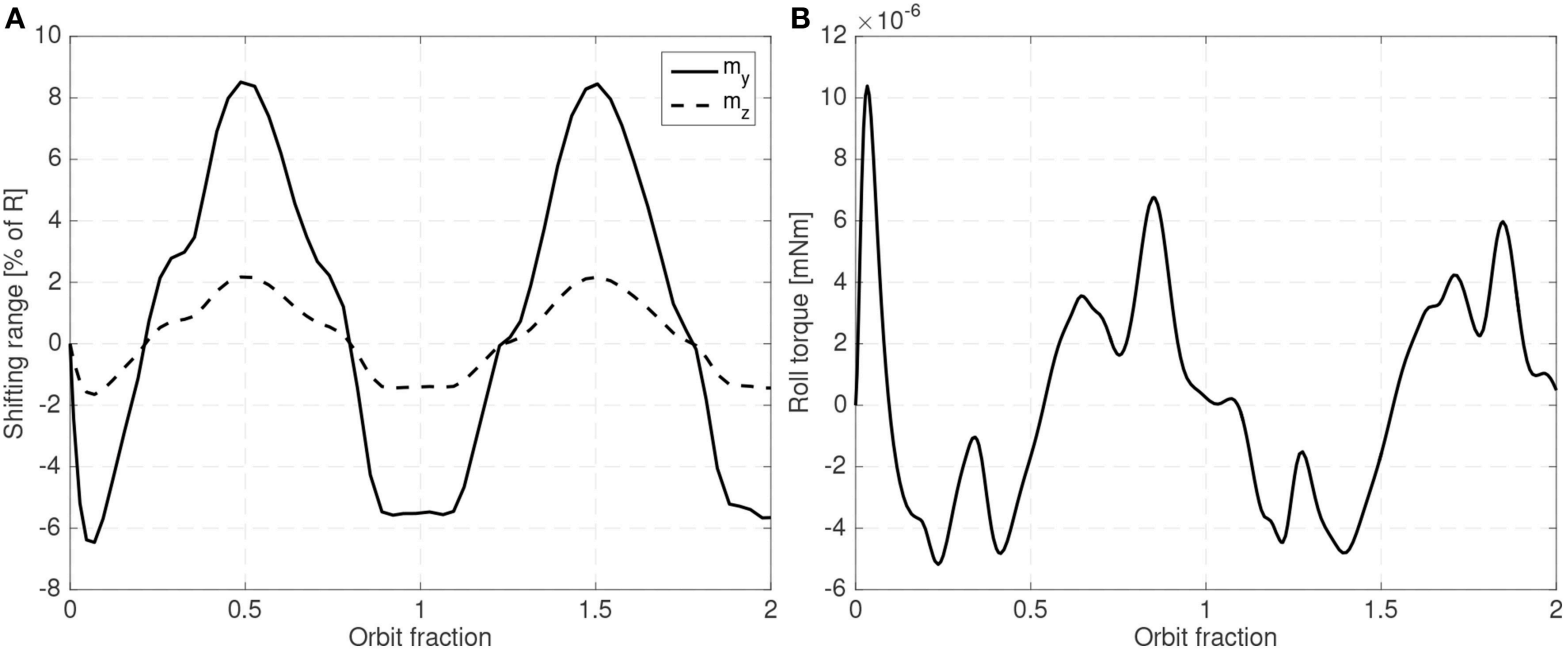
Shifting **(A)** and roll torque **(B)** required for an LQR controller.

As in all LQR based controllers, the weight matrices have to be carefully adjusted to obtain the desired combination of attitude error, shifting mass range and sufficiently small gains (so that the requirements on the sensors can be met). Figures 12, 13 show a steady state example of the evolution of the attitude, the shifting masses displacement and roll torque when an LQR controller is used and when the gravity gradient perturbation is also included.

A 25 cm spherical satellite operating at a 300 km Sun-Synchronous and with 6% of the mass allocated to the shifting masses (3% for each mass) and an estimated distance between the CoP and the host vehicle CoM of 3% of the radius have been used. In this case the spacecraft is operating in the unstable orientation (CoP leading the CoM). Uncertainty in the drag coefficient *C*_*D*_ has also been included. Although the controller is based on the linearized equations of motion, in the simulator the full equations of motion have been used. As seen in Figures 12, 13 the controller is able to maintain the system stable with small shifting masses and shifting range.

### 6.3. Quaternion Feedback With Partial Feedback Linearization

A more general approach that does not rely on linearization uses the well known quaternion feedback with a partial feedback linearization approach (Wie and Barba, 1985; Wie et al., 1989). The estimated aerodynamic torque τ_aero_ is also used to help with the feedback linearization but the terms related the shifting masses motion are left out as they are assumed to be negligible. Again, many of the assumptions are carried over.

- *Quat. Feedback Controller Asm.1*: Shifting masses are point masses.
- *Quat. Feedback Controller Asm.2*: The mass and inertia properties of the host vehicle and of the shifting masses are known.
- *Quat. Feedback Controller Asm.3*: The relative position, velocity and accelerations of the shifting masses are known.
- *Quat. Feedback Controller Asm.4*: Shifting masses velocities and accelerations have negligible effects on the dynamics.
- *Quat. Feedback Controller Asm.5*: Constant system inertia.
- *Quat. Feedback Controller Asm.6*: Constant atmospheric density.
- *Quat. Feedback Controller Asm.7*: Relative flow velocity matches the spacecraft's inertial velocity.

Under this control law the requested torque can be written as in Equation (53).

(53) $$\begin{array}{l} \tau_{\text{req}}=-K_{p}Jq_{e}-K_{d}J\omega_{\text{e}}+\omega_{0}\times J\omega_{0}-\tau_{\text{aero}} \end{array}$$

In Equation (53) ***q***_*e*_ denotes the vector elements of the error quaternion (Wie and Barba, 1985; Wie et al., 1989), ***ω***_e_ denotes the angular velocity error (for a stable attitude with respect to the orbit frame a pitch that matches the orbital motion needs to be included), ***J*** is the inertia matrix and the ***K***_*p*_ and ***K***_*d*_ diagonal matrices being the proportional and derivative gains in each axis respectively.

Some guidance to select the gains can be obtained by assuming small angles, a single degree-of-freedom, and that the shifting mass motion dynamics effects are negligible. In that case, the system reduces to a second order system and thus the proportional ***K***_*p*_ and derivative gains ***K***_*d*_ for each axis (*k*_*p*_ and *k*_*d*_) can be related to the desired closed loop natural frequency ***ω***_*n*_ and a damping ratio ξ as shown in Equation (54) (Wie et al., 1989).

(54) $$\begin{array}{l} k_{p}=2\omega_{n}^{2}\;k_{d}=2\xi \omega_{n} \end{array}$$

The quaternion feedback is particularly suited to be used when large attitude misalignments are present and thus it will be employed here for a detumbling scenario. If it is assumed that the system is composed by three shifting masses each moving along the body *B*_0_ roll $\hat{i}$, pitch $\hat{j}$ and yaw $\hat{k}$, the aerodynamic torque provided by the shifting masses can be written as follows (see Equation 17).

(55) $$\begin{array}{l} \tau_{\text{sm}}=\frac{F_{\text{aero}}}{M+m_{1}+m_{2}+m_{3}}\times \left[\begin{array}{c} m_{1}r_{1}\\ m_{2}r_{2}\\ m_{3}r_{3} \end{array}\right] \end{array}$$

With *m*_1_, *m*_2_, and *m*_3_ denoting the shifting masses and *r*_1_, *r*_2_ and *r*_3_ their shifting ranges along $\hat{i}$, $\hat{j}$ and $\hat{k}$ respectively. Note how Equation (55) is the last term of Equations (16, 17).

As the controller has no information about the actual direction and magnitude of the aerodynamic force, an estimate ***F***_aero_ needs to be used in the steering logic. A relative flow matching the inertial velocity and a mean atmospheric density are used to obtain this estimate. It is clear from Equation (55) that the shifting masses aerodynamic torque is perpendicular to the ***F***_aero_ and thus other actuators should provide the required torque that is parallel to ***F***_aero_.

The shifting masses position to achieve the requested perpendicular torque can be obtained using (Equation 56). Note that (Equation 56) is equivalent to the expression used to drive the magnetic torquers (replacing the magnetic field with the aerodynamic force ***F***_aero_ and the magnetic moment with the *mr* product).

(56) $$\begin{array}{l} \left[\begin{array}{c} m_{1}r_{1}\\ m_{2}r_{2}\\ m_{3}r_{3} \end{array}\right]=\frac{\tau_{req}\;\times \;F_{\text{aero}}\left(M\;+\;m_{1}\;+\;m_{2}\;+\;m_{3}\right)}{F_{\text{aero}}\cdot F_{\text{aero}}} \end{array}$$

The resulting shifting masses provided torque ***τ***_sm_ is just the component perpendicular to the flow direction and then additional actuators need to provide the parallel torque. To keep it consistent with past numerical examples only two shifting masses shifting along the body's *B*_0_ pitch $\hat{j}$ or yaw $\hat{k}$ axes will be used (thus *r*_1_ = 0). These couple of shifting masses will be complemented by a single ideal actuator acting along the *B*_0_ roll $\hat{i}$ axis.

- *Quat. Feedback Controller Asm.8*: Two shifting masses moving along the $\hat{j}$ and $\hat{k}$ axes.
- *Quat. Feedback Controller Asm.9*. Ideal roll actuator to partially provide the required torque parallel to ***F***_aero_.

**Figure 14:**
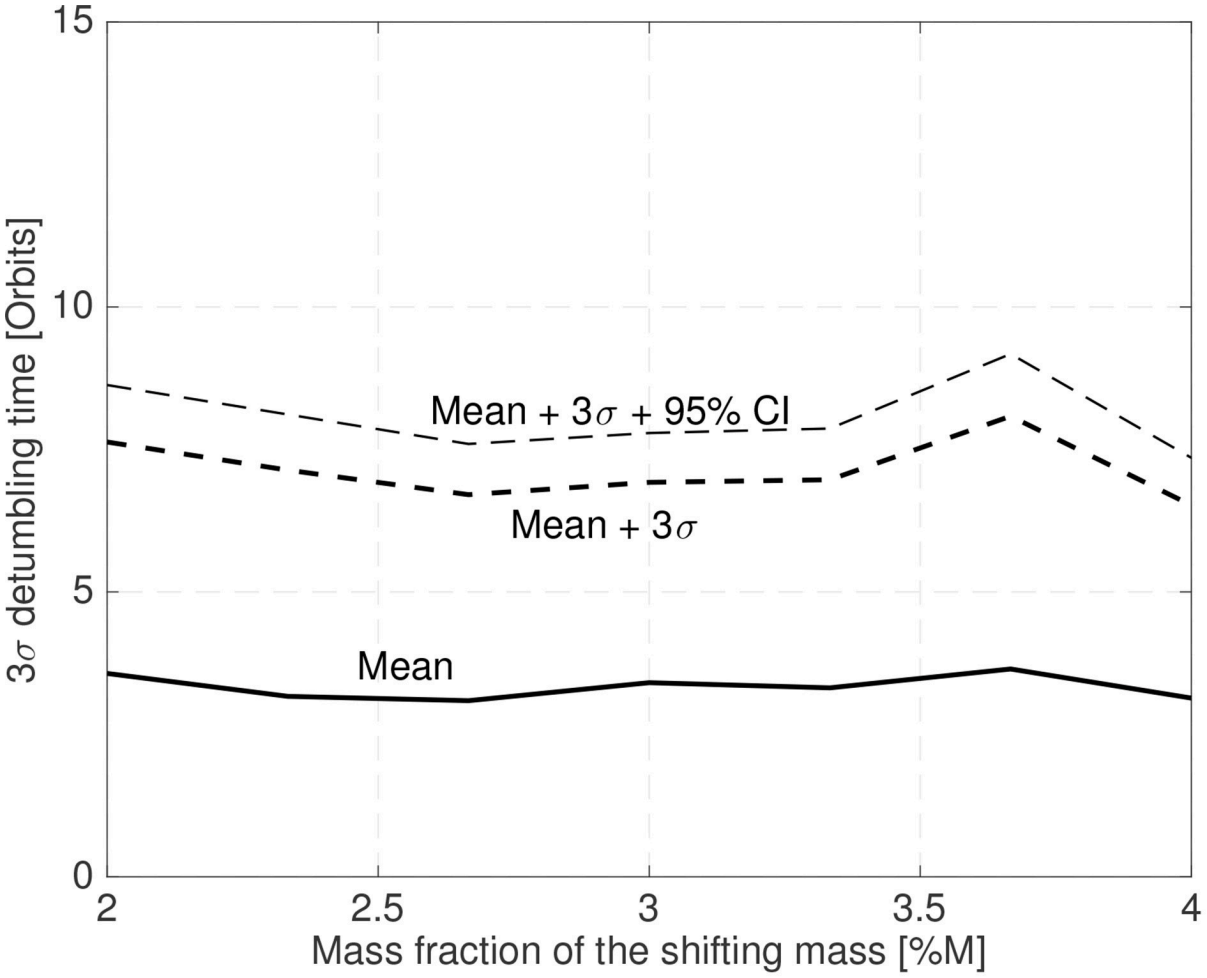
3σ maximum stabilization time for a 25 cm radius spacecraft in a 400 km altitude orbit.

This configuration has been used to stabilize a 25 cm spacecraft with a random initial attitude and an initial angular velocity in a random orientation with a magnitude between ±0.5deg/s (chosen with a random uniform distribution). Figure 14 shows the mean and the 3σ maximum stabilization time obtained by a 25 sample MonteCarlo simulation. The control law gains have been set according to Equation (54) with a bandwidth of twice the spacecraft natural frequency and ξ = 0.7. The shifting masses represent 6% of the host vehicle mass (3% each shifting mass) and the CoM leads the CoP by 3% of the spacecraft radius.

## 7. Practical Implementation on the Shift-Mass Sat 3U CubeSat

Small spacecraft are more sensitive to aerodynamic disturbances due to their high area to inertia ratio. A CubeSat operating at low altitude is thus a good first candidate to implement the proposed aerodynamic disturbance rejection method. This implementation exercise using a CubeSat, which are highly constrained platforms in terms of mass and volume, also serves as a practical feasibility check of the whole concept.

**Figure 15:**
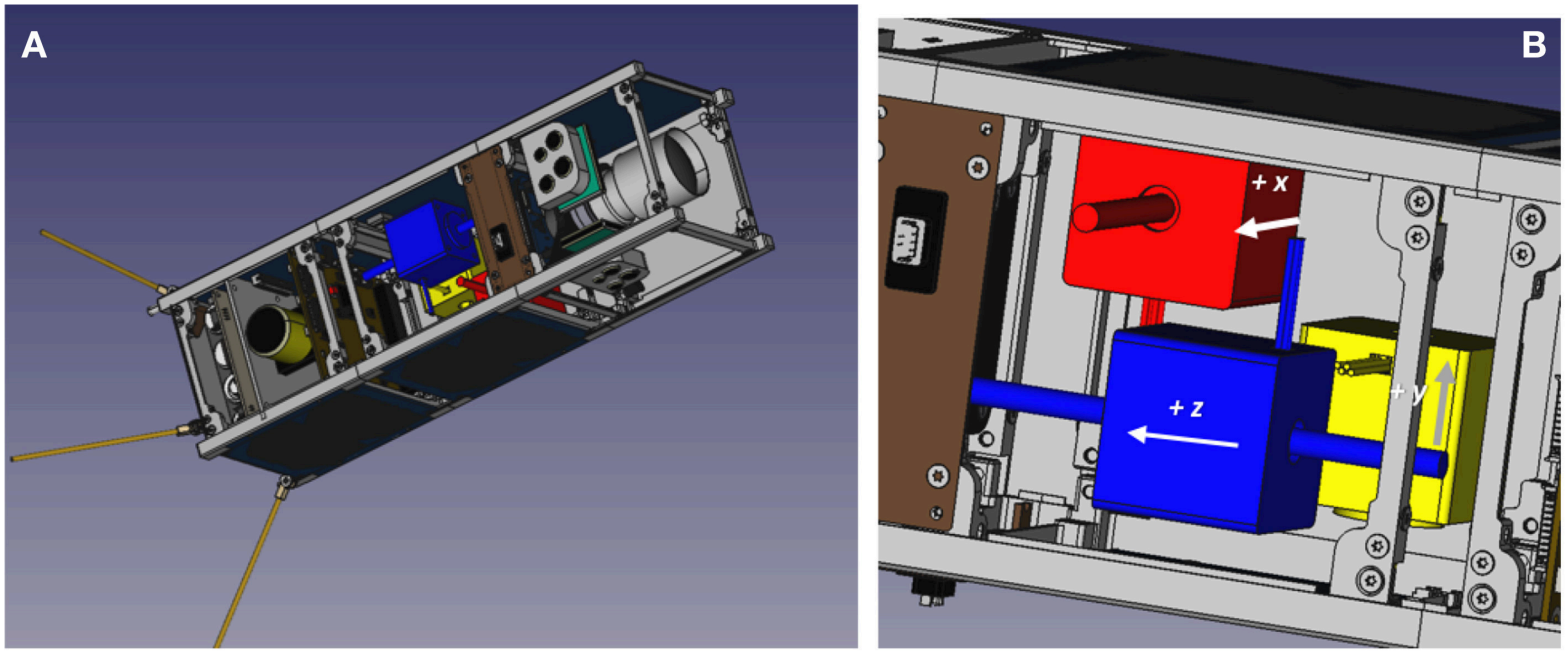
Prototype 3U CubeSat design with shifting masses **(A)** and detail of the three orthogonal shifting masses **(B)**.

A preliminary design of the “Shift-Mass Sat” 3U CubeSat with three orthogonal shifting masses is shown in Figure 15. All the components, subsystems and shifting masses, are COTS to ensure their commercial availability. More detailed information on this design can be found in Polat (2016).

The three 150 g shifting masses approximately take 75% of a 1U volume and have a 70 mm useful travel range. Magnetic torquers augment the shifting masses and complete the actuator set.

The performance of this design, paired with an LQR controller, has been evaluated using a numerical simulation. The CoM position, mass, inertia, shifting mass and travel range, magnetic dipole moment of the magnetic torquers, and aerodynamic properties used for the simulation have been derived from the prototype design. It is worth pointing that for this particular design, the shifting masses have a 1.82 mm control authority on the combined system's CoM position to modulate the aerodynamic torque direction and magnitude.

**Figure 16:**
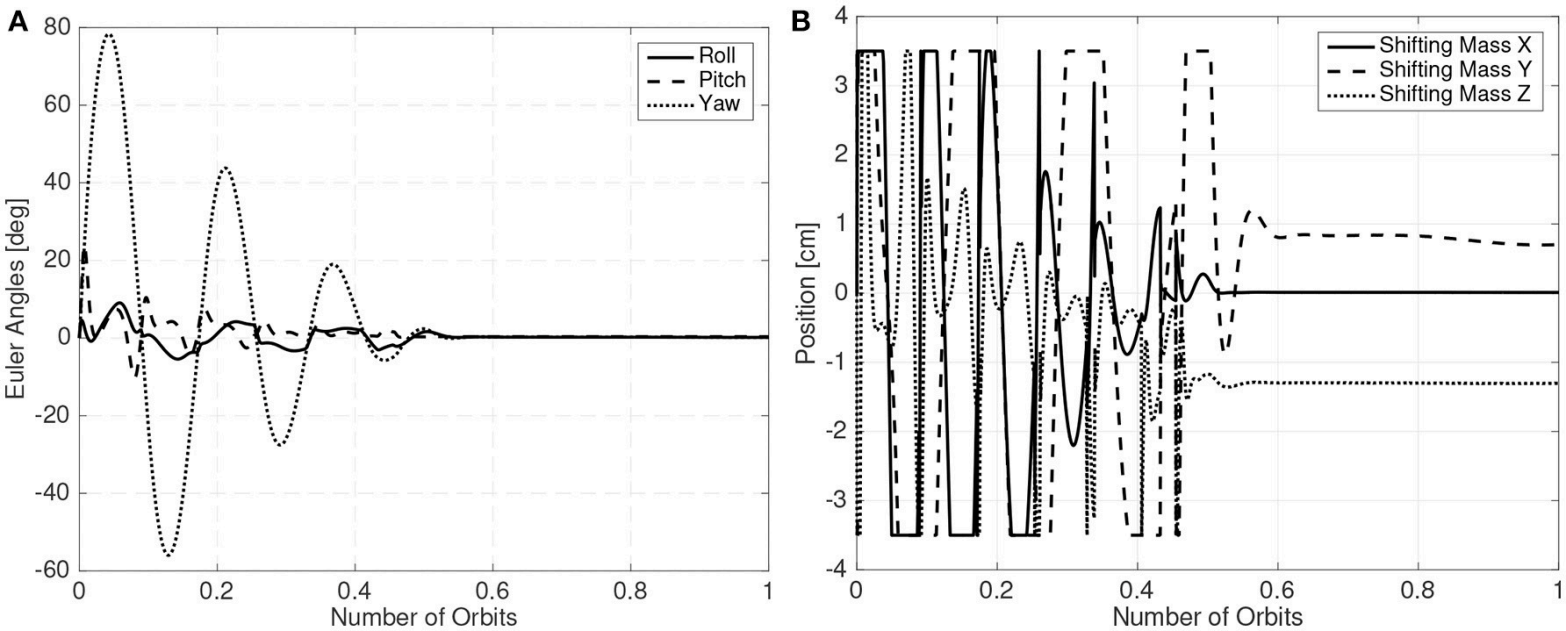
Euler angles **(A)** and shifting masses positions **(B)**.

The LQR controller is used for detumbling and to keep the spacecraft stable. A gain scheduling scheme, where less aggressive gains are employed during the detumbling phase is employed. The initial angular velocity of the CubeSat is chosen as 0.01 rad/s in all axis and the orbit altitude is set at 300 km. The shifting masses movement and Euler angles of one of these simulations are presented in Figure 16 showing the feasibility of the proposed method.

It is also worth noting that in Figure 16B the shifting masses exhibit a bias when the spacecraft is stabilized. This is because the CubeSat center of mass is not completely centered around the roll axis.

## 8. Conclusions

In conclusion, using a set of shifting masses that shift the spacecraft's center-of-mass is a viable method to reject the aerodynamic disturbances present at Very Low Earth Orbit. Despite the highly non-linear dynamics of a spacecraft with internal moving parts simple controllers based on the linearized equations of motion suffice to keep the spacecraft stable. The requirements imposed on the attitude determination subsystem and the shifting masses (shifting range and mass fraction) are well within practical limits. Achieving stabilization from arbitrary initial attitude and small angular velocities is also possible. A prototype implementation on a 3U CubeSat only using Commercial-Off-the-Shelf components and an Linear Quadratic Regulator controller demonstrates its technological feasibility.

Further research could be directed to develop other types of controllers, specially non-linear controllers, to drive the shifting masses in order to increasing the performance of the system. However, rigorously proving their stability can be a challenging endeavor.

## Author Contributions

JV-L and HP did most of the research. JV-L wrote the manuscript. MR supervised the research.

### Conflict of Interest Statement

The authors declare that the research was conducted in the absence of any commercial or financial relationships that could be construed as a potential conflict of interest.
